# Identification of QTN and Candidate Gene for Seed-flooding Tolerance in Soybean [*Glycine max* (L.) Merr.] using Genome-Wide Association Study (GWAS)

**DOI:** 10.3390/genes10120957

**Published:** 2019-11-21

**Authors:** Zheping Yu, Fangguo Chang, Wenhuan Lv, Ripa Akter Sharmin, Zili Wang, Jiejie Kong, Javaid Akhter Bhat, Tuanjie Zhao

**Affiliations:** 1 National Center for Soybean Improvement, Key Laboratory of Biology and Genetics and Breeding for Soybean, Ministry of Agriculture, State Key Laboratory of Crop Genetics and Germplasm Enhancement, Nanjing Agricultural University, Nanjing 210095, China; yu2016us@163.com (Z.Y.); 2017201054@njau.edu.cn (F.C.); 2017801220@njau.edu.cn (W.L.); ripa.sharmin@gmail.com (R.A.S.); njauwang@126.com (Z.W.); 2012094@njau.edu.cn (J.K.); javid.akhter69@gmail.com (J.A.B.); 2 Key Laboratory of Molecular Genetics, Guizhou Academy of Tobacco Science, Guiyang 550081, China

**Keywords:** soybean, genome-wide association study, seed-flooding tolerance, candidate gene, qRT-PCR

## Abstract

Seed-flooding stress is one of the major abiotic constraints severely affecting soybean yield and quality. Understanding the molecular mechanism and genetic basis underlying seed-flooding tolerance will be of greatly importance in soybean breeding. However, very limited information is available about the genetic basis of seed-flooding tolerance in soybean. The present study performed Genome-Wide Association Study (GWAS) to identify the quantitative trait nucleotides (QTNs) associated with three seed-flooding tolerance related traits, viz., germination rate (GR), normal seedling rate (NSR) and electric conductivity (EC), using a panel of 347 soybean lines and the genotypic data of 60,109 SNPs with MAF > 0.05. A total of 25 and 21 QTNs associated with all three traits were identified via mixed linear model (MLM) and multi-locus random-SNP-effect mixed linear model (mrMLM) in three different environments (JP14, HY15, and Combined). Among these QTNs, three major QTNs, viz., *QTN13*, *qNSR-10* and *qEC-7-2*, were identified through both methods MLM and mrMLM. Interestingly, *QTN13* located on Chr.13 has been consistently identified to be associated with all three studied traits in both methods and multiple environments. Within the 1.0 Mb physical interval surrounding the *QTN13*, nine candidate genes were screened for their involvement in seed-flooding tolerance based on gene annotation information and available literature. Based on the qRT-PCR and sequence analysis, only one gene designated as *GmSFT* (*Glyma.13g248000*) displayed significantly higher expression level in all tolerant genotypes compared to sensitive ones under flooding treatment, as well as revealed nonsynonymous mutation in tolerant genotypes, leading to amino acid change in the protein. Additionally, subcellular localization showed that *GmSFT* was localized in the nucleus and cell membrane. Hence, *GmSFT* was considered as the most likely candidate gene for seed-flooding tolerance in soybean. In conclusion, the findings of the present study not only increase our knowledge of the genetic control of seed-flooding tolerance in soybean, but will also be of great utility in marker-assisted selection and gene cloning to elucidate the mechanisms of seed-flooding tolerance.

## 1. Introduction

Soybean [*Glycine max* (L.) Merr] is one of the most economically important crops, and is rich in edible protein and oil. However, in recent years the changing global climate has resulted in increased occurrence of flooding events [1]. Due to the transient and excessive rainfall or irrigation with poor drainage, flooding stress has severely disrupted the crop growth and development, leading to considerable losses in grain yields globally. The most visible symptoms of flooding-stress include leaf chlorosis, necrosis, stunting, defoliation and plant death. However, to overcome the challenges of flooding stress, plants have evolved different mechanisms of physiological and morphological adaptations, including the formation of adventitious roots, aerenchyma development in roots, enhanced ethylene production and up-regulation of genes associated with leaf photosynthesis, ROS-scavenging and anaerobic metabolism [2,3,4]. However, the potential adaptive mechanisms involved in plants for responding to flooding stress are not well known. Previous study has reported that soybean is relatively susceptible to flooding stress at different growth stages such as germination, vegetative and reproductive stages [5], and flooding stress is a major limiting factor for normal soybean growth and grain yield. For example, flooding stress has been reported to reduce overall soybean yield by 17%–43% at the vegetative growth stage, and 50%–56% at the reproductive stage [6]. Although flooding stress tolerance has been widely studied in soybean at vegetative and reproduction stages, little attention has been focused on the germination stage. Hence, efforts are needed to understand the genetic mechanism and genetic basis underlying the seed-flooding tolerance at germination stage in soybean.

Flooding tolerance is a complex quantitative trait governed by multiple/polygenes genes, and is highly affected by environmental factors [7]. Extensive efforts have been made to identify the quantitative trait loci (QTLs) for flooding tolerance at different stages in soybean. Till now, 27 QTLs associated with flooding tolerance in soybean have been documented in Soybase (http://www.soybase.org). For example, one QTL associated with flooding tolerance on Chr.18 was identified at R1 growth stage that contributed to improved plant growth and grain yield [8]. In addition, previous study has detected two QTLs associated with flooding tolerance on Chr.05 and Chr.13 using two RIL populations [9], accounting for 10% and 16% of the phenotypic variation, respectively. Moreover, seven QTLs viz., *ft1* to *ft7* for flooding tolerance were detected at early vegetative growth stage in soybean [10]. Among them, *ft1* located on Chr.06 exhibited the largest phenotypic effects and explained 30.5%–49.2% of the phenotypic variation in two different environments. Furthermore, six putative QTLs were identified to be associated with flooding tolerance score (FTS) and flooding yield index (FYI) traits on Chr.11 and Chr.13 [11]. Interestingly, these QTLs appeared to be overlapped or closely linked with the previously mapped QTLs [9,10]. Although extensive QTLs for flooding tolerance have been detected, most of these QTLs were mainly identified either at vegetative or reproductive stages. Limited information is available about QTLs associated with seed-flooding tolerance at germination stage in soybean. It is known that seed germination is a critical phase that determines the successful establishment and productivity of soybean in water-logged soils. So far, only four QTLs associated with germination rate (GR) and normal seedling rate (NSR) under seed-flooding stress have been reported at the germination stage [12]. Among these four QTLs, *sft2* located on Chr.08 displayed the largest effect on seed-flooding tolerance and it was potentially involved in seed coat pigmentation. 

Although many QTLs associated with flooding tolerance in soybean have been reported at different growth stages, most of these loci were identified via linkage mapping with a relatively low genomic resolution. Thus, these QTLs possess very low selection accuracy, and cannot be effectively utilized in marker-assisted selection (MAS) for breeding enhanced flooding tolerance in soybean. Conventional linkage mapping is based on segregating populations derived from bi-parental crosses [13], resulting in limited recombination events and poor resolution of linkage mapping. Hence, it is difficult for bi-parental segregating populations to detect tightly associated markers and some minor alleles per locus. In contrast to classical linkage mapping, genome-wide association study (GWAS) using natural population based on linkage disequilibrium (LD) exhibited high mapping resolution and abundant genetic variation due to the high ancestral recombination events in natural populations [14]. Thus, GWAS is an efficient method to detect single nucleotide polymorphisms (SNPs) or quantitative trait nucleotide (QTNs) associated with important complex quantitative traits, and predict or identify causal genes [15,16]. In recent years, GWAS has been widely used for association mapping to dissect the genetic architecture of complex traits in various plants, including *Arabidopsis thaliana* [17], rice [18], maize [19], wheat [20], cotton [21], and soybean [22]. However, the GWAS mapping for seed-flooding tolerance in soybean has not been reported, to date. Among all the methods used for GWAS analysis, the mixed linear model (MLM) is the most commonly used method in association mapping [23]. Meanwhile, some advanced MLM-based methods have been subsequently developed such as compressed MLM (CMLM), which was proposed to save the computing time and improve the statistical efficiency. However, these methods are based on single-locus genome-wide scanning, and Bonferroni correction for multiple tests is still required. Recently, one better method was proposed called multi-locus random-SNP effect MLM (mrMLM), which is based on the combination of single locus genome scanning and multiple locus model [24]. The mrMLM exhibited significantly improved power and accuracy in quantitative trait nucleotide (QTN) detection and effect estimation. In the present study, both MLM and mrMLM methods are used to identify the SNPs/QTNs, as well as candidate genes associated with seed-flooding tolerance at germination stage in soybean.

By keeping the above into view, the objectives of our study were to elucidate the underlying genetic mechanisms of seed-flooding tolerance in soybean using the natural variation. In the present study, we utilized 60,109 high-quality SNPs to identify significant QTNs associated with seed-flooding tolerance in soybean by GWAS strategy as well as to identify underlying possible candidate genes. These findings will not only enhance our understanding of the genetic mechanisms underlying seed-flooding tolerance in soybean at germination stage, but will also be highly useful in marker-assisted breeding (MAB) for developing soybean varieties with improved seed-flooding tolerance.

## 2. Materials and Methods

### 2.1. Plant Materials

In the present study, we used a Yangtze-Huai soybean breeding germplasm (YHSBG) population that consists of 347 diverse soybean genotypes for GWAS analysis, and this population was provided by the National Center for Soybean Improvement, Nanjing, Jiangsu Province, China. Seeds of the whole population were harvested from two different environments Jiangpu Experimental Station (abbreviated as JP) of Nanjing Agricultural University in Nanjing (latidude 32.12°N; longitude 118.37°E), China, in 2014; and the Experimental Farm of Huaiyin Institute of Agricultural Sciences (abbreviated as HY) in Huaian (latidude 33.31°N; longitude 119.01°E), China, in 2015. The JP14 and HY15 represent two different environments. All lines of the YHSBG population were planted in a complete randomized block design (CRBD) with three replications in each environment. The list of 347 soybean lines was presented in Supplementary Table S1. 

### 2.2. Seed-Flooding Tolerance Evaluation

Fifty healthy and good-quality seeds were selected, and sterilized with 70% ethanol for 10 seconds. These seeds were further rinsed with distilled water for three times. Furthermore, seeds were subjected to submergence treatment/seed-flooding stress by dipping them in 350 mL plastic cups containing 100 mL distilled water at 25 °C that were covered by sterilized petri dishes placed on the top of cups to prevent loss of water through evaporation. The submergence treatment was applied for different time intervals (0, 2, 3, 4, 5, 6 and 7 days) to determine the optimum duration of flooding treatment. The experiment was conducted in a completely randomized design (CRD). All the lines were phenotypically evaluated for three traits related to seed-flooding tolerance at germination stage, viz., germination rate (GR), normal seedling rate (NSR) and electric conductivity (EC). For the estimation of EC, we used conductivity meter (model: DDS-307A) to record the EC value of steep-water in the plastic cups. Germination experiment was carried out by paper rolling method as follows: stressed seeds were grown for 5 days under normal conditions, and the number of germinated seeds and normal seedlings were recorded. Seeds with radicle length more than 1 cm were regarded as the germinated. Seedlings with normal cotyledon and radicle were regarded as the normal seedlings. For control, seeds without submergence treatment were grown under the same normal conditions. The soaking and germination experiment was conducted in the lab at 25 °C with a long-day light cycle (16 h light/8 h dark). The GR and NSR were estimated using the following formula:

GR = (germination rate under flooding treatment)/(germination rate in control) × 100%

NSR = (normal seedling rate under flooding treatment)/(normal seedling rate in control) × 100%

### 2.3. Phenotypic Data Analysis

Descriptive statistics such as mean, range, standard deviation (SD), skewness and kurtosis were calculated for the selected YHSBG population in two different environments JP14 and HY15 using SAS PROC UNIVARIATE programs. Analysis of variance (ANOVA) and Pearson’s correlation coefficient among traits related to seed-flooding tolerance were estimated using SAS PROC generalized linear model (GLM) and PROC CORR programs, respectively [25]. Broad-sense heritability (*h*^2^) of the association mapping panel was estimated as:

$$h^{2}=\sigma_{G}^{2}/(\sigma_{G}^{2}+\sigma_{GE}^{2}/n+\sigma_{e}^{2}/\mathrm{nr})$$

for combined environments, and

$$h^{2}=\sigma_{G}^{2}/(\sigma_{G}^{2}+\sigma_{e}^{2})$$

for an individual environment, where $\sigma_{G}^{2}$ represents the genotypic variance, $\sigma_{GE}^{2}$ is the variance of the genotype-by-environment interaction, $\sigma_{e}^{2}$ is the error variance, n is the number of environments, and r represents the number of replications within each environment [26].

### 2.4. SNP Data Analysis 

Restriction-site-associated DNA sequencing (RAD-seq) approach was utilized in the present study to sequence the genomic DNA of all lines of YHSBG population, and this sequencing was carried by Beijing Genomics Institution (BGI), Shenzhen, China. First, the genomic DNA of all 347 soybean lines was extracted from young leaves using a modified CTAB method [27]. *Taq* I enzyme was used to digest this genomic DNA for constructing genomic DNA library. The DNA fragments of 400–700 bp were selected and sequenced using an Illumina HiSeq 2000 standard protocol for multiplexed shotgun genotyping (MSG), and 90-mer paired-end reads were generated [28]. All the sequence reads were aligned to the reference genome of the Glyma.Wm82.a1.v1.1 [29] using the SOAP2 software [30]. Based on the Bayesian estimation of the site frequency, RealSFS was utilized for the SNP calling [31]. The SNP data was screened at a rate of missing and heterozygous allele calls ≤30% and then the missing genotypes were imputed using fastPHASE software [32]. A total of 60,109 SNPs with minor allele frequencies (MAF) > 5% for 347 lines were selected from 87,308 SNPs and used for the GWAS analysis.

### 2.5. Population Genetic Analysis

In this study, 3851 SNPs were screened using the indep-pairwise command option of pLINK software [33]. Model-based cluster analysis was performed to infer genetic structure and to define the number of clusters (gene pools) in the dataset using the software STRUCTURE version 2.2 [34]. The number of presumed populations (*K*) was set from 1 to 10, and the analysis was repeated four times, then each Q and the related *P*-value were calculated. The most likely number of subpopulations was determined by the Delta *K* method [35]. Neighbor-joining tree was constructed using TASSEL 5.0 software [36], then principal component analysis (PCA) was performed using the *R* package software. In addition, Kinship was calculated using TASSEL 5.0, and LD between pairwise SNPs was estimated with the squared correlation coefficient *r*^2^ using the RTM-GWAS V1.1 software [37]. The LD decay rate was measured as the chromosomal distance where the *r*^2^ dropped to half of its maximum value.

### 2.6. Genome-Wide Association Analysis

In the present study, 60,109 SNPs with minor allele frequencies (MAF) > 5% were utilized for the genotyping of YHSBG population consisting of 347 soybean accessions. To validate and increase the accuracy of the GWAS results, we used two different models MLM and mrMLM for association analysis in this study. Besides, the population structure (Q) and kinship (*K*) matrix were analyzed. The mixed linear model (MLM) was performed using the TASSEL 5.0 software, and in this model Q and K matrices act as a fixed effect and random effect, respectively. However, multi-locus random-SNP-effect mixed linear model (mrMLM) was performed by the R package mrMLM V2.1 [24]. The Bonferroni threshold *P* = 1.0 × 10^−4^ (−log_10_ *P* = 4.0) was used as the critical threshold to identify significant SNP-trait association/quantitative trait nucleotides (QTNs). With the −log_10_ *P* ≥ 4.0 of significance level, the significant SNPs identified by GWAS were analyzed by Haploview4.2, and the QTNs were identified by LD-block analysis [38].

### 2.7. Candidate Gene Predictions and Expression Analysis

Based on the gene annotations available at the SoyBase (http://www.soybase.org) and Phytozome (http://phytozome.jgi.doe.gov) databases, as well as in the available literature, we predicted possible candidate genes within the major QTN region identified on Chr.13 in the present study. These candidate genes were further subjected to qRT-PCR analysis to verify their differential expression between contrasting soybean genotypes. The root parts of seedlings were selected for RNA extraction and qRT-PCR analysis. Total RNA was extracted using the RNA Simple Total RNA kit (TIANGEN, Beijing, China). The cDNA was synthesized using the Prime ScriptTM RT Reagent Kit (TaKaRa, Shiga, Japan) following a standard protocol. Then qRT-PCR assay was performed to examine the differential expression levels of the candidate genes in selected lines using the Bio-rad CFX96 system (Bio-Rad, Hercules, USA). The PCR conditions were 95 °C for 3 min followed by 37 cycles of 95 °C for 15 s, 55 °C for 30 s and 72 °C for 30 s. Polymerase chain reaction were normalized using the *Ct* value corresponding to the soybean actin gene (*Actin11*) as an internal control. Three biological replications were used, and three measurements were performed on each replicate. All the primers used were designed by Vector NTI 11.5 (Supplementary Table S2).

### 2.8. Sequence Analysis of Candidate Genes

To investigate the nucleotide mutation of candidate genes related to seed-flooding tolerance in seed-flooding tolerant and sensitive lines, the sequence analysis of the CDS region and 2 kb promoter region of these genes was performed. Total RNA was extracted using the RNA Simple Total RNA kit (TIANGEN, China). The reverse transcription was conducted using Transcript two-step gDNA Removal and the cDNA was synthesized by Prime ScriptTM RT Reagent Kit (TaKaRa, Japan). Additionally, the CDS of these candidate genes were amplified using Phanta^®^ Max Super Fidelity DNA Polymerase from Vazyme (Supplementary Table S2). The band size of PCR products was verified using agarose gel electrophoresis (3%). PCR samples were sent to Generalbiol for sequencing. The nucleotide sequence and amino acid sequence alignment was carried out using DNAMAN and BioXM2.6 software, respectively.

### 2.9. Plasmid Construction and Subcellular Localization 

The coding sequence of *GmSFT* (*Glyma.13g248000*) was introduced into *pJRH0641-GFP* to generate the *pJRH0641-GmSFT::GFP* vector. Primers were designed according to the nucleotide sequence of *GmSFT* and the *XhoI* restriction site on the *pJRH0641-GFP* (Supplementary Table S2). The construction of the recombinant plasmid was conducted using In-Fusion^®^ HD Cloning Plus (TaKaRa, Japan). Then the recombinant plasmid was transformed into *E.coli* DH5α, and finally transformed into *Agrobacterium tumefaciens* strain *EHA105* by electroporation method. The transient expression was carried out through the infection of *Agrobacterium* liquids containing the recombinant plasmid in young *N. benthamiana* leaves. At 36 h following infection, the fluorescence in *N. benthamiana* leaves was detected using a confocal laser scanning microscope (Zeiss LSM780, Oberkochen, Germany). 

## 3. Results 

### 3.1. Determination of Optimum Seed-Flooding Treatment Duration

**Figure 1:**
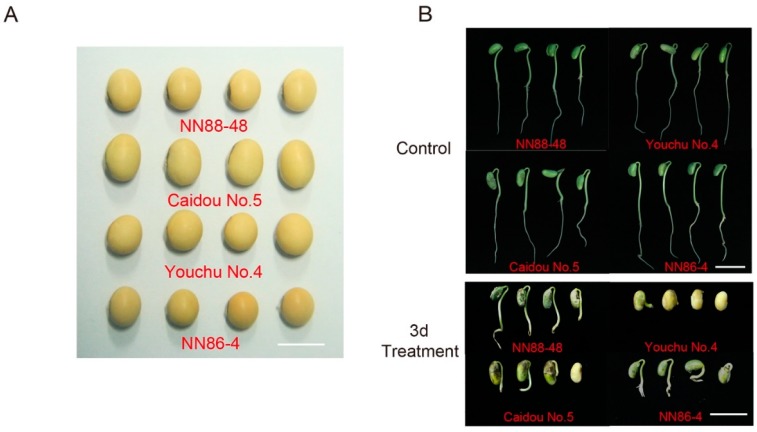
Germination performance of four core lines NN88-48, Caidou No.5, NN86-4 and Youchu No.4. (**A**) Grain shape of four core lines. (**B**) The germination and growth of seedlings among selected four lines under control and three days treatment. Scale bars are 1.0 cm in (**A**) and 3.0 cm in (**B**).

To determine the optimum seed-flooding treatment duration to be used for seed-flooding tolerance evaluation in soybean association mapping population, we tested the germination performance of four core lines viz., Nannong88-48(NN88-48), Caidou No.5, Nannong86-4(NN86-4) and Youchu No.4 (included in the YHSBG population) under different flooding treatment intervals (0, 2, 3, 4, 5, 6 and 7 days). These four soybean lines are all cultivated genotypes, and displayed similar grain shape, including seed size and seed coat color (Figure 1A). According to the previous results of optimum seed-flooding tolerance evaluation in our lab, the seed-flooding stress was performed under three days treatment [39]. Interestingly, in this experiment, the growth performance of seedlings after 3 days flooding treatment revealed significantly considerable difference among these selected four lines (Figure 1B). Furthermore, the analysis of variance (ANOVA) displayed significant differences of both GR and NSR among these four lines under 3 days treatment (Supplementary Table S3). Hence, by considering the above results, the 3 day seed-flooding treatment was considered the optimum seed-flooding treatment duration for the evaluation of seed-flooding tolerance of population in the present study.

### 3.2. Phenotypic Evaluation

The seed-flooding tolerance of 347 soybean lines of YHSBG population was evaluated using three germination-related traits GR, NSR and EC. The values of descriptive statistics, ANOVA (*F*-value) and estimates of heritability (*h^2^*) for all three studied traits of the YHSBG population in two different environments JP14 and HY15 were presented in Table 1. In JP14, the mean of GR and NSR were 0.55 and 0.40, respectively, and their ranges were 0–1.00 and 0–0.94. Furthermore, the mean of EC was 1248 us/mL, and ranges from 153 to 2840 us/mL. However, compared with the mean in JP14, the means of GR and NSR in HY15 were lower, whereas the mean of EC was relatively higher (Table 1), suggesting that seed-flooding tolerance in soybean is an environmentally sensitive trait. The results of *F* values from ANOVA revealed highly significant differences across genotypes/lines (G) and environments (E). Moreover, significant line × environment (G×E) interaction was observed for EC in this study (Table 1). Furthermore, the heritability estimate (*h*^2^) of GR, NSR and EC across both environments were high and ranged from 0.69 to 0.77, indicating that most of the phenotypic variance of seed-flooding tolerance in the association mapping population was genetically controlled.

### 3.3. Genetic Diversity, Population Structure and Linkage Disequilibrium Analysis

**Figure 2:**
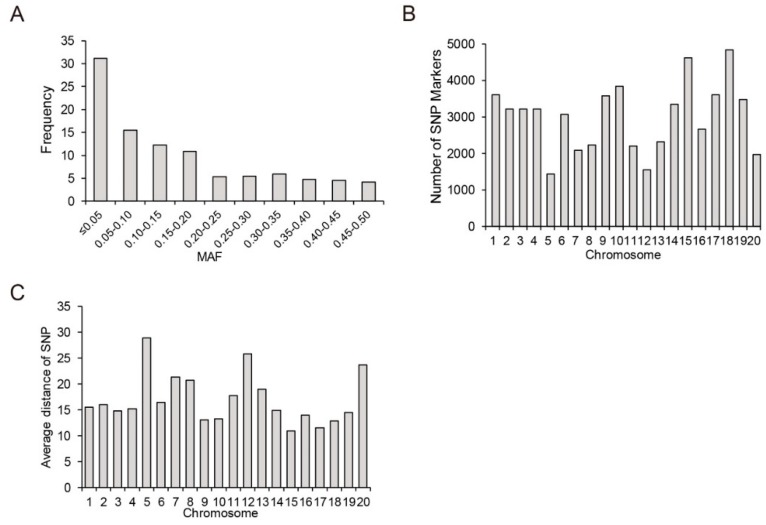
Distribution analysis of 60,109 SNPs across 347 lines. (**A**) Distribution of minor allele frequency (MAF). (**B**) Distribution of SNPs on 20 chromosomes. (**C**) Average distance of SNPs on 20 chromosomes.

In the present study, out of a total 87,308 SNPs, 31.15% of the SNPs with the minor allele frequency (MAF) ≤ 0.05 were excluded for further analysis, whereas the remaining 60,109 SNPs with MAF > 0.05 were utilized to analyze the genetic diversity in the YHSBG association mapping population of 347 soybean lines (Figure 2A). These selected SNPs were unevenly distributed on 20 different chromosomes of soybean (Figure 2B). The average number of SNPs on each chromosome was 3005, with the minimum number of 1432 SNPs on Chr.05 and the maximum number of 4836 SNPs on Chr.18. Moreover, the average distance between SNPs on 20 different chromosomes were shown in Figure 2C, which ranged from 10.96 kb on Chr.15 to 28.90 kb on Chr.05.

**Figure 3:**
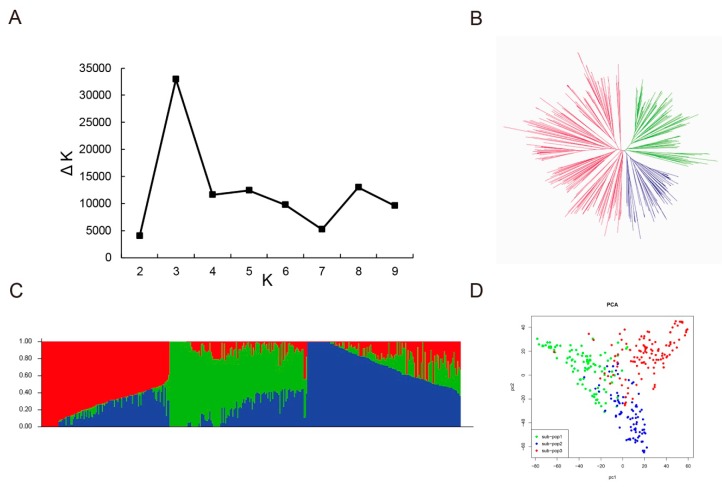
Population structure analysis of 347 soybean lines. (**A**) Calculation of the true *K* value in the association population. (**B**) A maximum likelihood neighbor-joining tree of the tested 347 lines. (**C**) Population structure estimated by STRUCTURE. Three colors represent three subpopulations. Each vertical column represents one individual and each colored segment in each column represents the percentage of the individual in the population. (**D**) PCA plots of the 347 lines.

Furthermore, three different methods—Bayesian model-based methods, principal component analysis (PCA) and phylogenetic analysis—were used to determine the population structure of 347 soybean lines of YHSBG population. Delta *K* (∆*K*) was calculated using STRUCTURE 2.3.4 (Figure 3A; *K* = 1–10), and exhibited the presence of three subpopulations (selected *K* = 3) based on ∆*K* values (Figure 3C). Furthermore, the phylogenetic tree and PCA analysis displayed the consistent results in agreement with the population structure analysis (Figure 3B,D).

**Figure 4:**
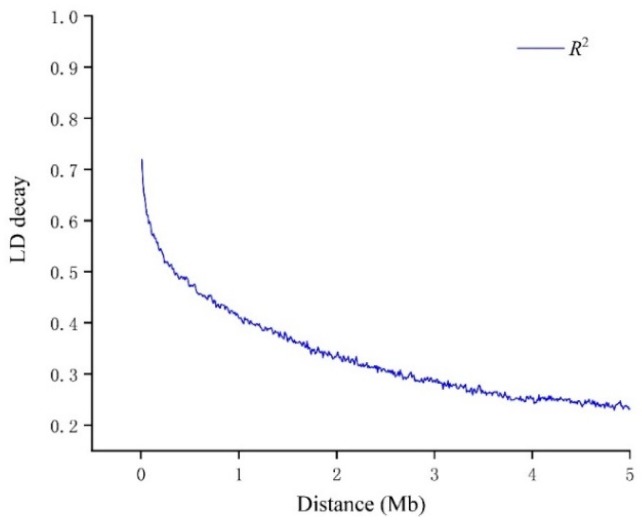
Genome-wide average linkage disequilibrium (LD) decay in the population of 347 soybean lines. The mean LD decay rates were estimated by the squared correlation coefficient (*R*^2^).

In the present study, we estimated the *r^2^* values of all pairs of SNPs located within 10 Mb of each other and determined the linkage disequilibrium (LD) decay trend based on regression to the negative natural logarithm. In comparison with LD decay of other plant species such as *Arabidopsis thaliana* [40], rice [41] and maize [42], soybean displayed relatively high level of LD [43]. In this study, *r^2^* decreased gradually with increased distance, and the LD decay distance was estimated at ~1.60 Mb, where *r*^2^ dropped to half of its maximum value (Figure 4).

### 3.4. GWAS Analysis via MLM

**Figure 5:**
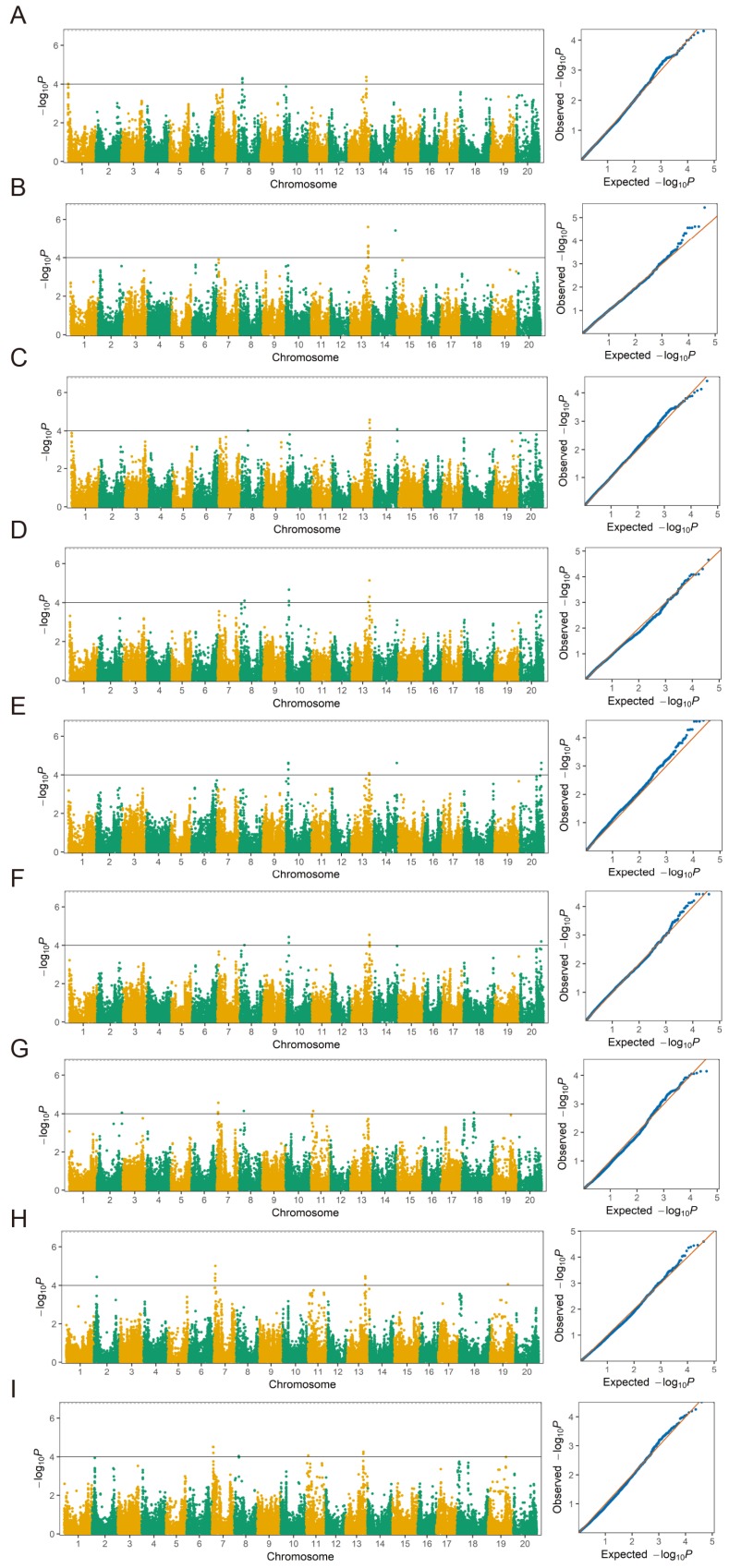
Genome-wide association study utilized in the population, including Manhattan plots and quantile-quantile (QQ) plots for (**A**) GR-JP14. (**B**) GR-HY15. (**C**) GR-Combined. (**D**) NSR-JP14. (**E**) NSR-HY15. (**F**) NSR-Combined. (**G**) EC-JP14. (**H**) EC-HY15. (**I**) EC-Combined.

In the present study, the MLM model was performed to identify significant QTNs associated with GR, NSR and EC. A total of 8, 6 and 11 QTNs were identified to be associated with GR, NSR and EC, respectively, at the significance level of −log_10_*P* = 4 in JP14, HY15 and Combined-environments (Table 2 and Figure 5). The eight QTNs of GR were distributed on four different chromosomes Chr.01, Chr.08, Chr.13 and Chr.14. Among these QTNs, only *qGR-13-2* located on Chr.13 was detected consistently in all three environments, explaining 4.51%–6.62% of the phenotypic variation (PV). Furthermore, this QTN were significantly associated with SNP marker *Gm13_35324537*, and displayed the largest effect of PV = 6.62% in HY15. Additionally, *qGR-13-3* and *qGR-14* were identified in two different environments, and these two QTNs accounted for 4.35%–6.38% of the PV. The remaining five QTNs has been identified in only one environment (Table 2). For NSR, a total of six QTNs were detected and located on five different chromosomes Chr.08, Chr.10, Chr.13, Chr.14 and Chr.20 (Table 2). Among them, *qNSR-10*, *qNSR-13-1* and *qNSR-13-2* were identified in all three environments, and were considered as the stable QTNs associated with NSR. Furthermore, these three QTN explained 4.81%–5.38%, 4.43%–6.02% and 4.37%–4.69% of the PV, respectively (Table 2). In addition, two QTNs related to NSR viz., *qNSR-8* and *qNSR-20* were detected in two different environments, explaining 4.38%–4.52 % and 4.76%–5.32% of the PV, whereas the remaining *qNSR-14* was only identified in single environment. For EC, we identified 11 QTNs through MLM approach, and these QTNs were distributed on seven different chromosomes viz., Chr.02, Chr.07, Chr.08, Chr.11, Chr.13, Chr.18 and Chr.19 (Table 2). Out of these QTNs, only *qEC-7-2* was consistently identified in all three environments (JP14, HY15 and Combined), explaining 4.75%–5.87% of the PV (Table 2), and this QTN was significantly associated with the same SNP marker *Gm07_2942021* on Chr.07. Furthermore, *qEC-8*, *qEC-11* and *qEC-13-1* were detected in two different environments, explaining 4.37%–4.62%, 4.76%–4.81% and 4.67%–4.95% of the PV, respectively. The remaining seven QTNs associated with EC were only identified in a single environment, and accounted for 4.38%–5.10% of the PV.

### 3.5. GWAS Analysis via mrMLM, and Comparative Analysis of MLM and mrMLM Results

To confirm the reliability of the QTNs identified by MLM method and detect more QTNs associated with GR, NSR and EC, a multi-locus random effect MLM (mrMLM) method was used to conduct GWAS analysis. As shown in Table 3, a total of eight, seven and six QTNs associated with GR, NSR and EC were detected via mrMLM (Table 3). For GR, one QTN *qGR-13-2* associated with marker *Gm13_35324537* was consistently identified in all three studied environments, and this QTN displayed large effects with PV ranged from 5.44%–7.22%. The remaining seven QTNs of GR were only detected in a single environment, explaining 3.96%–7.17% of the PV. In the case of the NSR trait, seven QTNs were detected and distributed on seven different chromosomes. Among these QTNs, only *qNSR-13* was identified in all environments and exhibited large effects. However, *qNSR-10* was detected in two environments (JP14 and HY15). Moreover, the remaining five QTNs for NSR were detected in single environments. For EC, a total of six QTNs were identified, accounting for 4.55–6.81% of the PV. Out of these QTNs associated with EC, two QTNs *qEC-7* and *qEC-13* were detected in two different environments (JP14 and HY15). However, the remaining four QTNs *qEC-3*, *qEC-10*, *qEC-12* and *qEC-19* were only identified in single environment (Table 3).

In summary, a total of 25 and 21 QTNs associated with all three traits related to seed-flooding tolerance were identified via MLM and mrMLM, respectively (Table 2 and Table 3). For MLM, five major QTNs, viz., *qGR-13-2*, *qNSR-10*, *qNSR-13-1*, *qNSR-13-2* and *qEC-7-2* were detected in all three environments. Among them, both *qGR-13-2* and *qNSR-13-1* were significantly associated with SNP marker *Gm13_35324537* and designated as *QTN13*. Although *qEC-13-1* was only detected in two environments (HY15 and Combined), this QTN (*QTN13*) was also closely linked with *Gm13_35324537*. As expected, for mrMLM, one major QTN associated with *Gm13_35324537* for all traits (*qGR-13-2*, *qNSR-13-1* and *qEC-13-1*) was identified, indicating that this critical QTN (*QTN13*) on Chr.13 can be detected by both MLM and mrMLM. Furthermore, *qNSR-10* and *qEC-7-2* were also identified by both MLM and mrMLM (Table 3). Nevertheless, the *qNSR-13-2* identified by MLM could not be verified by mrMLM. Based on the above results, we obtained one major and stable *QTN13* associated with *Gm13_35324537* for all traits, and this QTN was identified in all environments as well as in both MLM and mrMLM models. Thus, this major *QTN13* on Chr.13 was potentially utilized as the hotspot region for fine mapping and candidate gene analysis of seed-flooding tolerance in soybean.

### 3.6. Candidate Gene Prediction and qRT-PCR Analysis

**Figure 6:**
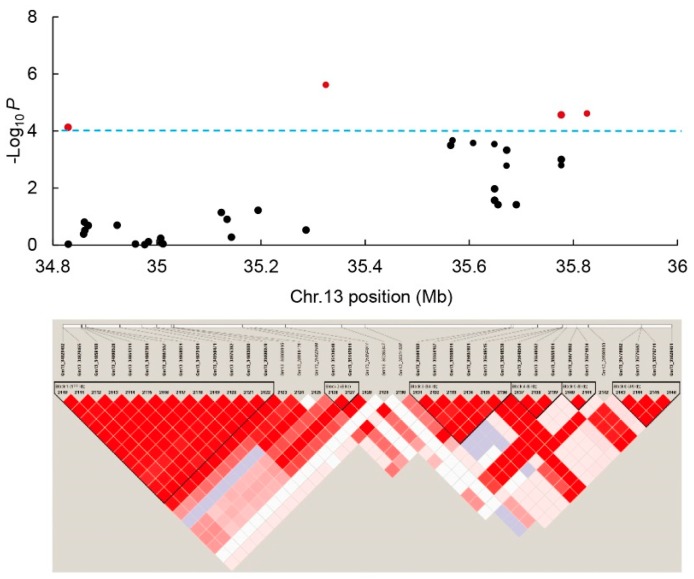
Haplotype blocks and candidate regions of the genome significantly associated with identified major QTNs for GR on Chr.13 in the association population.

**Figure 7:**
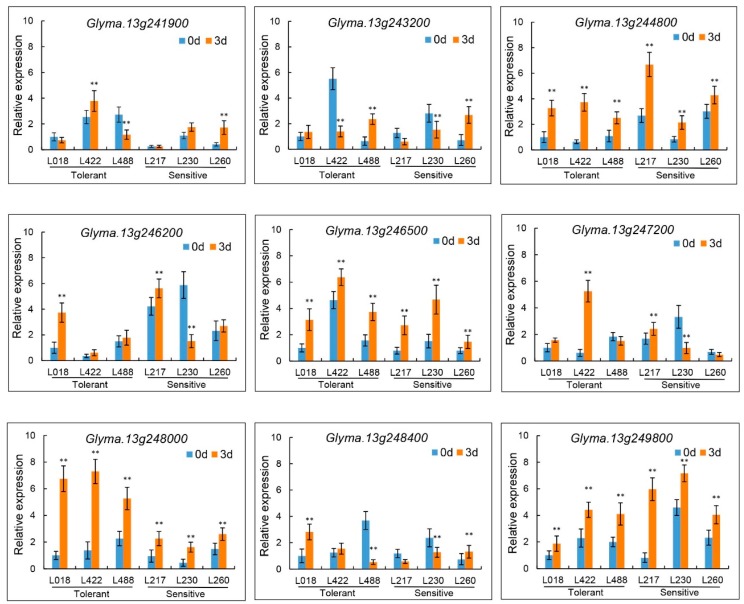
Relative expression levels of nine candidate genes associated with seed-flooding tolerance in tolerant genotypes (L018, L422, and L488) and sensitive genotypes (L217, L230, and L260) under 3 d flooding treatment.

As the stable *QTN13* located on Chr.13 was consistently identified to be associated with all three studied traits by both methods as well as in multiple environments, we performed the candidate gene prediction analysis in the genomic region surrounding the *QTN13*. Based on the LD distance, we extended and selected the region of 500 kb upstream and downstream of the peak SNP marker *Gm13_35324537/QTN13* on both sides, and a total of 131 genes were located within this 1.0 Mb region. Moreover, the haplotype block analysis of all SNPs present in this 1.0Mb candidate region was conducted using Haploview 4.2 software (Figure 6), and a total of 37 SNPs were included in this candidate region (Supplementary Table S4). Based on the gene functional annotations from the Phytozome (https://phytozome.jgi.doe.gov) and Soybase (http://www.soybase.org) databases and the available literature, nine candidate genes were predicted from these 131 genes for possibly regulating the seed-flooding tolerance in soybean (Table 4). To further investigate the expression levels of these nine candidate genes under flooding treatment, we conducted the qRT-PCR analysis. Based on the evaluation of 347 lines of association panel for seed-flooding tolerance in two different environments using GR, NSR and EC parameters, three seed-flooding tolerant lines (L018, L422, and L488) and three seed-flooding sensitive lines (L217, L230 and L260) were screened, and utilized to examine the differential expressions of these above nine genes (Supplementary Table S5). Four candidate genes *Glyma.13g244800*, *Glyma.13g246500*, *Glyma.13g248000* and *Glyma.13g249800* exhibited relatively high expression levels in all six lines after 3d of flooding treatment compared with control, suggesting that these four genes were induced under flooding stress treatment (Figure 7). Among these four genes, only *Glyma.13g248000* displayed relatively higher expression levels in tolerant lines (L018, L422, and L488) than that in sensitive lines (L217, L230, and L260), which suggested that *Glyma.13g248000* was probably involved in the seed-flooding tolerance in soybean.

### 3.7. Sequence Analysis and Subcellular Localization of GmSFT 

**Figure 8:**
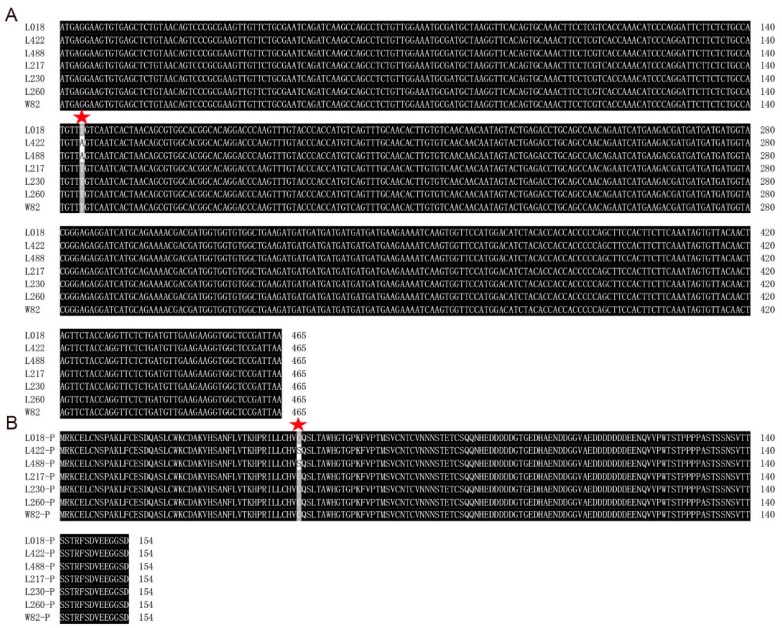
Nucleotide sequence and amino acid sequence alignment of *Glyma.13g248000* in seed-flooding tolerant (L018, L422, and L488) and sensitive (L217, L230, and L260) genotypes. (**A**) The nucleotide sequence alignment. (**B**) Amino acid sequence alignment. W82 (William82), represents the reference genome of W82 (*Glycine max*).

Based on the results of qRT-PCR analysis, the relative expressions of four candidate genes (*Glyma.13g244800*, *Glyma.13g246500*, *Glyma.13g248000* and *Glyma.13g249800*) were induced under 3d flooding stress treatment. To further clarify the nucleotide mutation/polymorphism in these four candidate genes, we carried out the sequence analysis. By comparing the nucleotide differences of these genes between seed-flooding tolerant and sensitive lines, *Glyma.13g248000* revealed one base mutation (T–A) at 145bp position in two seed-flooding tolerant lines L422 and L488, and this nonsynonymous mutation resulted in the single amino acid change (Cys-Ser) in protein (Figure 8). However, no base mutations/polymorphisms were observed in the other three candidate genes between tolerant and sensitive lines. Furthermore, sequence analysis of the 2 kb promoter region upstream of these four genes was performed. However, no nucleotide mutation was observed among these six tolerant and sensitive genotypes. Although no base mutation of *Glyma.13g248000* was identified at 145 bp position in the seed-flooding tolerant line (L018), but the confirmation of this mutation in the remaining two seed-flooding tolerant line L422 and L488 suggested that *Glyma.13g248000* was the most possible candidate gene associated with seed-flooding tolerance identified in this study, and this gene was designated as *GmSFT*.

**Figure 9:**
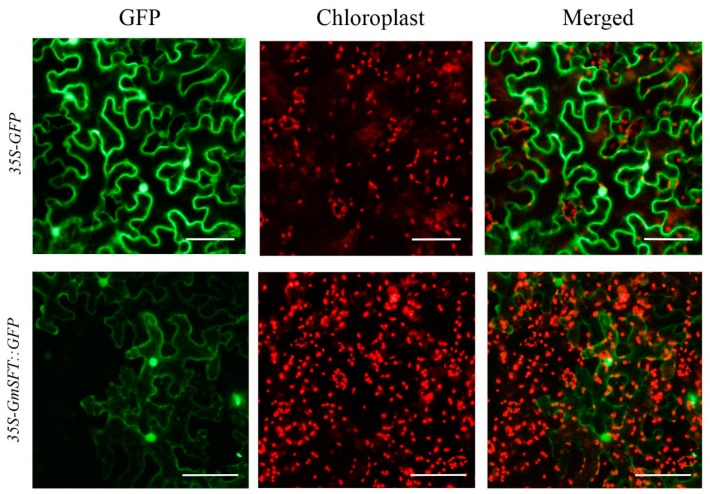
Diagram showing subcellular localization of *GmSFT* gene in the leaf of *N. benthamiana*. Bar indicates 50 μm.

To further explore the localization of *GmSFT*, we performed the subcellular localization of *GmSFT* by generating the recombinant plasmid *35S-GmSFT::GFP*, which were transiently transformed in *N. benthamiana* leaves using *Aagrobacterium*-mediated transformation. Based on the online prediction of subcellular localization via SoftBerry(http://linux1.softberry.com/), *GmSFT* was probably localized in the extracellular region. However, based on wet lab experiment, as shown in Figure 9, the *35S-GFP* (as control) was located in the nucleus, membrane and portions of cytoplasm. However, the green fluorescence of *35S-GmSFT::GFP* was mainly distributed in the nucleus and cell membrane rather than in the extracellular region at 36h of post-transfection, which confirmed that *35S-GmSFT::GFP* was localized in the nucleus and plasma membrane. 

## 4. Discussion

Plants, being sessile, are subjected to various abiotic stresses, among which flooding stress has received increased attention in recent decades as it severely inhibits plant growth and development, and in turn affects the grain yields and agricultural production. Hence, achieving soybean varieties with improved flooding tolerance was one of primary goals in soybean breeding programs [44]. As an economically important crop, soybean is relatively susceptible to flooding stress at early germination stage. Seed germination is an important growth stage that determines crop establishment and final crop yields. Besides, seed germination is accompanied by various metabolic activities and physiological changes [45]. In this context, GR and NSR are the two most important parameters that can be used to evaluate seed germination of crop species, and hence were utilized in the present study. Furthermore, strong positive correlations of *r* = 0.90 in JP14 and *r* = 0.92 in HY15 (*P* < 0.001) were observed between GR and NSR in the YHSBG association population (Supplementary Table S6). Previous study has reported that flooding stress resulted in the intracellular seed substance leakage due to rapid water imbibition [12]. Therefore, to better evaluate the seed-flooding tolerance of soybean, we measured the electric conductivity (EC) of this association population. The results of correlation analysis revealed that EC was negatively correlated with both GR and NSR. Overall, the comprehensive analysis of GR, NSR and EC is an effective way for the evaluation of seed-flooding tolerance at germination stage.

So far, several studies have been conducted to reveal the genetic basis and underlying genetic mechanism of flooding tolerance in soybean, and many QTLs associated with flooding tolerance have been detected in multiple environments [8,9,10,12]. However, most of these QTL mapping studies were carried out using linkage mapping analysis based on low-density genetic maps. It is clear that flooding tolerance is a complex quantitative trait governed by multiple minor genes, and is highly influenced by environmental factors. Hence, most of the earlier reported QTLs were not both stable and confirmed, and therefore had not been successfully utilized in marker-assisted selection (MAS) for breeding soybean cultivars with enhanced flooding tolerance. To overcome these limitations of linkage mapping, a linkage disequilibrium (LD)-based genome-wide association study (GWAS) was used in the present study. The earlier studies proved that the application of GWAS is an alternative and powerful strategy to precisely detect and define the genomic position of loci/genes associated with complex traits [46,47]. The LD is the non-random association of alleles at two or more different loci. However, the degree of genome-wide LD plays an important role in the application of GWAS, and it is affected by many forces, including relatedness, population stratification, genetic drift, and linkage [48]. Moreover, the extent of LD is one of the critical factors determining the resolution of association mapping [49]. In this study, the LD decay of the soybean genome was approximately estimated at 1.6Mb, which was substantially higher than other plants, such as rice (75 kb in *indica*) [41], *Arabidopsis thaliana* (10 kb) [40] and cultivated sunflower (1.1 kb) [50]. Previous studies have revealed that the typical self-pollinated crops such as soybean showed relatively higher LD level than that of cross-pollinated species [51,52]. Furthermore, based on the resequencing of 31 soybean genomes, Lam et al. (2010) demonstrated that both wild and cultivated soybeans displayed high LD, whereas the LD level of cultivated soybean was higher [43]. Additionally, in the present study the average *Fst* value of the GWAS population was 0.1707, which was low, as expected. Hence, both the high LD and low *Fst* value indicated the low genetic diversity of this GWAS population [53], and this can be explained by the fact that majority of the genotypes of GWAS population has common parentage, which was derived from the crosses among less than 80 domestic or exotic cultivated soybean elite lines. Moreover, the same geographical origin, viz., the Yangtze-Huai valley, of most of these genotypes also contributed to the low genetic diversity of this GWAS population. Hence, an effective way of improving the genetic diversity of the GWAS population would be to select the genotypes with various genetic background (including cultivars, landraces and wild species) from different geographical regions. In the present study, the RADseq strategy was utilized for the sequencing of whole GWAS population. The RADseq approach uses restriction enzymes to cut genomic DNA into small fragments, and then these short fragments are sequenced [54]. However, compared with whole genome resequencing (WGRS), the coverage of RADseq is low, and the overall coverage of soybean genome in this study was 4.57%. Presently, the cost of WGRS is still high to re-sequence the large population, hence till the WGRS become cost-effective and feasible, targeted sequencing seems to be more cost-effective option for large scale marker discovery and association mapping, particularly in case of large sized genomes such as soybean [55]. Thus, RADseq is considered to be a fast and economical strategy for population genetic studies within relatively low budgets [56]. In addition, previous study demonstrated that RADseq is an effective way for population genotyping by detecting thousands of polymorphisms [57]. The average number of SNPs on each chromosome (3005) and the average distance between SNPs (15.75 kb) in this study also indicate relatively rich SNPs detected by RADseq for GWAS. 

For the GWAS analysis, we used two different methods of MLM and mrMLM to identify the QTNs associated with seed-flooding tolerance. By using the genotypic data of 60,109 SNPs with MAF > 0.05, a total of 25 and 21 QTNs associated with all three traits (GR, NSR and EC) were identified via MLM and mrMLM at the significance threshold (–log_10_*P* = 4) in YHSBG association population (Table 2 and Table 3). Among these QTNs, four stable QTNs, viz., *QTN13*, *qNSR-10*, *qNSR-13-2,* and *qEC-7-2*, were detected in all environments (JP14, HY15, and Combined) via MLM, explaining 4.37%–6.62% of the phenotypic variation (PV), whereas three QTNs (*QTN13*, *qNSR-10*, and *qEC-7-2*) were identified by mrMLM. Interestingly, we identified one stable QTN (*QTN13*) on Chr.13 that was associated with SNP marker *Gm13_35324537* for all traits (*qGR-13-2*, *qNSR-13-1* and *qEC-13-1*), and this QTN was detected in all environments. Moreover, this stable *QTN13* on Chr.13 was successfully detected through mrMLM. As shown in Table 3, three major QTNs, viz., *qGR-13-2*, *qNSR-13-1* and *qEC-13-1* identified in all environments were consistently associated with *Gm13_35324537*. Previous study has reported one QTL related to flooding tolerance near marker *Satt269* on Chr.13 within the genetic interval of 16.08-23.56cM [9]. Based on the genomic sequence information of this QTL associated with *Satt269* from Soybase (http://www.soybase.org), this QTL was located in 15.31Mb of physical interval on Chr.13, but the physical position of *Gm13_35324537* was at 35.32Mb of Chr.13. Therefore, this stable *QTN13* identified on Chr.13 in our study is a newly QTN associated with seed-flooding tolerance in soybean. Although the other three stable QTNs viz., *qNSR-10*, *qNSR-13-2* and *qEC-7-2* identified via MLM were detected in all environments, all these three QTNs were only related to a single trait. Among them, *qNSR-13-2* associated with *Gm13_35776682* was mapped at the position of 35.78Mb on Chr.13, which was only 452 Kb away from *Gm13_35324537*, indicating that this region containing *qNSR-13-1* and *qNSR-13-2* on Chr.13 was potentially a hotspot possessing large effects on seed-flooding tolerance. The *qNSR-10* identified via MLM was also verified via mrMLM, and was tightly linked with marker *Gm10_4316320* at 4.30 Mb on Chr.10. Additionally, previous study reported two QTLs on Chr.10 related to flooding tolerance in soybean, which are located at the genetic intervals of 81.08–85.08 cM and 106.02–108.02 cM [10]. However, the corresponding physical position of these two QTLs was around 40.6 Mb and 44.7 Mb, suggesting that these two QTLs and *qNSR-10* were separated at considerable physical distance. Moreover, *qEC-7-2* was associated with *Gm07_2942021* and located at 2.94 Mb on Chr.07. Furthermore, a QTL on Chr.07 at the genetic distance of 72.37–74.37 cM was reported [10], with the corresponding physical position of 17.76 Mb. Thus, based on the above results, we can conclude that all these four stable QTNs identified in the present study were novel QTNs for seed-flooding tolerance in soybean, and most of them displayed large phenotypic effects.

To date, a total of 27 QTLs related to flooding tolerance in soybean have been documented in the Soybase (http://www.soybase.org). However, not all of these QTLs have been physically mapped, and no further efforts have been made to mine candidate genes for flooding tolerance underlying these genomic loci. In this study, we identified a highly stable *QTN13* on Chr.13 that was consistently associated with all three studied traits (GR, NSR, and EC). In addition, this *QTN13* displayed large phenotypic contribution and effects on seed-flooding tolerance. Therefore, we performed candidate gene prediction analysis within 1.0Mb region surrounded the QTN13/*Gm13_35324537*, and subsequently qRT-PCR analysis was used to validate the predicted candidate genes. Based on the functional annotations and predictions, a total of nine candidate genes directly or indirectly associated with flooding tolerance were screened (Table 4). The qRT-PCR results further revealed that four candidate genes out of above nine genes were significantly induced under seed-flooding stress treatment. Among them, *Glyma.13g248000* displayed relatively higher expression levels in all three tolerant genotypes (L018, L422 and L488) relative to three sensitive genotypes (L217, L230 and L260). Furthermore, sequence alignment analysis of these four candidate genes between tolerant and sensitive genotypes revealed that only *Glyma.13g248000* showed the nonsynonymous mutation in two seed-flooding tolerant lines (L422 and L488), which leads to single amino acid change (Cys-Ser) in protein. However, no mutations of *Glyma.13g248000* were observed in the tolerant line (L018), and this might be attributed to their different genetic background. In conclusion, *Glyma.13g248000* was considered to be the most likely possible candidate for gene regulating seed-flooding tolerance in soybean, and was named as *GmSFT*. Moreover, based on the wet lab experiment *GmSFT* was localized in the nucleus and cell membrane. 

Based on the functional annotation of *GmSFT*, it encodes a B-box type zinc finger protein. In rice, previous study has reported that a CCCH-type zinc finger was significantly induced by hypoxia stress (lack of oxygen) caused by water submergence, indicating the potential roles of zinc finger in flooding tolerance [58]. Moreover, several studies have also demonstrated that the B-box type zinc finger protein was involved in multiple abiotic stresses in plants. For example, as the transcription factor of B-box type zinc finger, *AtCOL4* plays critical roles in response to ABA and salt stress. One grape B-box ZFP family, *VvZFPL*, has been reported to show increased tolerance to cold, drought and salt stresses in transgenic *Arabidopsis* plants. Additionally, as a B-box type zinc finger protein, MdBBX10 has been revealed to exhibit enhanced tolerance to salt and drought stresses in *Arabidopsis* [59,60,61]. Therefore, all the above findings support that *GmSFT* might be the candidate gene regulating seed-flooding tolerance in soybean. However, further functional evidence and validation is still required to prove its actual involvement in governing the seed-flooding tolerance in soybean. 

## 5. Conclusions

In this study, we used the GWAS strategy to identify the QTNs associated with three traits related to seed-flooding tolerance GR, NSR and EC in the YHSBG population using genotypic data of 60,109 SNPs in soybean. A total of 25 and 21 QTNs associated with GR, NSR and EC were identified via MLM and mrMLM methods in three different environments (JP14, HY15, and Combined). Among these QTNs, four major and stable QTNs, viz., *QTN13*, *qNSR-10*, *qNSR-13-2*, and *qEC-7-2*, were detected via MLM in all three environments, and three of them (*QTN13*, *qNSR-10*, and *qEC-7-2*) were also identified via mrMLM. Based on the comparative analysis of MLM and mrMLM, the stable *QTN13* was reported to be associated with all three traits as well as has been verified in both methods and multiple environments. Subsequently, the 1.0 Mb region around *QTN13/Gm13_35324537* was used for candidate gene prediction as well as qRT-PCR analysis. Based on the results of qRT-PCR and sequence analysis, *GmSFT*(*Glyma.13g248000*) was considered to be the most likely candidate gene regulating seed-flooding tolerance in soybean. However, further functional validation is required to determine its roles in seed-flooding tolerance of soybean. In summary, these findings extended our understanding of the genetic basis of seed-flooding tolerance in soybean, and will be highly useful in breeding soybean varieties with enhanced seed-flooding tolerance through marker-assisted breeding as well as for gene cloning.

## Figures and Tables

**Table 1:** Descriptive statistics, heritability (*h*^2^) and *F* Values from ANOVA for germination rate (GR), normal seedling rate (NSR) and electric conductivity (EC) in two environments (JP14 and HY15).

Trait	Env.	Mean ± SD	Range	*h* ^2^	*F* values from ANOVA		
					Line	Env.	Line × Env.
GR	JP14	0.55 ± 0.29	0–1	0.74	9.84^***^	13.27^***^	0.47^ns^
	HY15	0.43 ± 0.26	0–0.95				
NSR	JP14	0.40 ± 0.27	0–0.94	0.69	8.11^***^	7.04^**^	0.37^ns^
	HY15	0.27 ± 0.24	0–0.93				
EC	JP14	1248 ± 446	154–2840	0.77	3.58^***^	5.19^**^	2.16^***^
	HY15	1309 ± 450	228–3620				

Env., represents environment; SD, represents standard deviation; JP14 and HY15, represent two environments Jiangpu 2014 and Huaiyin 2015; ** indicates significant level at *P* < 0.01; *** indicates significant level at *P* < 0.001; *h*^2^ broad sense heritability.

**Table 2:** Quantitative trait nucleotides (QTNs) associated with germination rate (GR), normal seedling rate (NSR) and electric conductivity (EC) via MLM.

Trait	QTN	Env.^a^	Chr.^b^	SNP	Position (bp)	*P* Value	−log_10_ *P*^c^	MAF	*R*^2^ (%)	Effect	Allele
GR	*qGR-1*	JP14	1	Gm01_380964	380964	9.83 × 10^−5^	4.01	0.07	4.49	-0.027	T/A
	*qGR-8-1*	JP14	8	Gm08_9896483	9896483	5.67 × 10^−5^	4.25	0.29	4.52	-0.097	C/T
	*qGR-8-2*	JP14	8	Gm08_10412475	10412475	9.37 × 10^−5^	4.03	0.25	4.26	-0.003	A/G
	*qGR-8-3*	Combined	8	Gm08_14242918	14242918	9.71 × 10^−5^	4.01	0.07	4.28	-0.253	G/T
	*qGR-13-1*	JP14	13	Gm13_34829465	34829465	7.42 × 10^−5^	4.13	0.09	4.48	-0.018	C/T
	*qGR-13-2*	JP14	13	Gm13_35324537	35324537	4.75 × 10^−5^	4.32	0.07	4.51	-0.257	G/T
	(*QTN13*)	HY15	13	Gm13_35324537	35324537	2.44 × 10^−6^	5.61	0.07	6.62	-0.285	G/T
		Combined	13	Gm13_35324537	35324537	2.65 × 10^−5^	4.58	0.06	4.97	0.197	C/T
	*qGR-13-3*	HY15	13	Gm13_35826401	35826401	2.43 × 10^−5^	4.61	0.05	5.28	-0.267	A/T
		Combined	13	Gm13_35776711	35776711	2.36 × 10^−5^	4.63	0.31	4.87	0.147	A/G
	*qGR-14*	HY15	14	Gm14_46766676	46766676	3.71 × 10^−6^	5.43	0.31	6.38	0.172	A/G
		Combined	14	Gm14_46766676	46766676	8.28 × 10^−5^	4.08	0.13	4.35	-0.175	C/T
NSR	*qNSR-8*	JP14	8	Gm08_9370630	9370630	7.92 × 10^−5^	4.1	0.12	4.38	-0.11	A/G
		Combined	8	Gm08_9370743	9370743	9.72 × 10^−5^	4.01	0.08	4.52	0.229	A/G
	*qNSR-10*	JP14	10	Gm10_4316320	4316320	2.17 × 10^−5^	4.66	0.08	5.38	0.242	A/G
		HY15	10	Gm10_4314632	4314632	2.65 × 10^−5^	4.58	0.08	5.26	0.213	A/T
		Combined	10	Gm10_4316320	4316320	3.73 × 10^−5^	4.43	0.07	4.81	-0.264	G/T
	*qNSR-13-1*	JP14	13	Gm13_35324537	35324537	7.18 × 10^−6^	5.14	0.07	6.02	-0.282	G/T
	(*QTN13*)	HY15	13	Gm13_35324537	35324537	8.20 × 10^−5^	4.09	0.05	4.43	-0.248	G/T
		Combined	13	Gm13_35324537	35324537	2.85 × 10^−5^	4.54	0.05	4.95	0.226	G/T
	*qNSR-13-2*	JP14	13	Gm13_35776682	35776682	5.02 × 10^−5^	4.3	0.06	4.69	-0.213	C/G
		HY15	13	Gm13_35826420	35826420	9.82 × 10^−5^	4.01	0.05	4.37	0.209	G/T
		Combined	13	Gm13_35826420	35826420	7.12 × 10^−5^	4.15	0.05	4.41	0.238	C/T
	*qNSR-14*	HY15	14	Gm14_46766676	46766676	2.45 × 10^−5^	4.61	0.31	5.31	0.146	A/G
	*qNSR-20*	HY15	20	Gm20_42414884	42414884	2.41 × 10^−5^	4.62	0.05	5.32	0.24	C/T
		Combined	20	Gm20_42414884	42414884	6.29 × 10^−5^	4.2	0.22	4.76	272.188	C/G
EC	*qEC-2-1*	HY15	2	Gm02_5181577	5181577	3.63 × 10^−5^	4.44	0.22	5.09	285.2	C/T
	*qEC-2-2*	JP14	2	Gm02_49796905	49796905	8.91 × 10^−5^	4.05	0.47	4.46	-90.75	C/T
	*qEC-7-1*	HY15	7	Gm07_2485335	2485335	3.10 × 10^−5^	4.51	0.09	5.1	-519.94	A/G
	*qEC-7-2*	JP14	7	Gm07_2942021	2942021	2.69 × 10^−5^	4.57	0.09	5.53	-465.45	A/G
		HY15	7	Gm07_2942021	2942021	9.74 × 10^−6^	5.01	0.09	5.87	-532.98	A/G
		Combined	7	Gm07_2942021	2942021	6.34 × 10^−5^	4.2	0.33	4.75	-106.07	A/G
	*qEC-8*	JP14	8	Gm08_9896483	9896483	7.20 × 10^−5^	4.14	0.29	4.62	184.03	C/T
		Combined	8	Gm08_9752396	9752396	9.25 × 10^−5^	4.03	0.16	4.37	310.06	A/G
	*qEC-11*	JP14	11	Gm11_4188194	4188194	7.25 × 10^−5^	4.14	0.16	4.81	326.88	A/G
		Combined	11	Gm11_4188194	4188194	8.70 × 10^−5^	4.06	0.13	4.76	247.19	A/G
	*qEC-13-1*	HY15	13	Gm13_35324537	35324537	3.47 × 10^−5^	4.46	0.08	4.95	324.5	G/T
	*(QTN13)*	Combined	13	Gm13_35324537	35324537	7.12 × 10^−5^	4.15	0.08	4.67	333	G/T
	*qEC-13-2*	HY15	13	Gm13_35648515	35648515	4.37 × 10^−5^	4.36	0.08	4.91	400.56	A/G
	*qEC-13-3*	HY15	13	Gm13_36352911	36352911	6.55 × 10^−5^	4.18	0.11	4.72	41.08	C/T
	*qEC-18*	JP14	18	Gm18_23932848	23932848	8.78 × 10^−5^	4.06	0.34	4.38	152.49	G/T
	*qEC-19*	HY15	19	Gm19_35610187	35610187	8.87 × 10^−5^	4.05	0.4	4.58	272.19	A/G

^a^ represents the different environments, JP14 represents Jiangpu2014, JP14 represents Huaiyin2015, Combined represents the Combined-environment based on JP14 and HY15; ^b^ represents the chromosome; ^c^ represents the negative log_10_ transformed *P*-value; MAF represents minor allele frequency.

**Table 3:** Quantitative trait nucleotides (QTNs) associated with germination rate (GR), normal seedling rate (NSR) and electric conductivity (EC) via mrMLM.

Trait	QTL	Env.^a^	Chr.^b^	SNP	Position (bp)	*P* Value	−log_10_ *P*^c^	LOD	MAF	*R*^2^ (%)	Effect	Allele	CIM^d^
GR	*qGR-2*	HY15	2	Gm02_46786754	46786754	4.14 × 10^−5^	4.38	3.58	0.47	4.74	0.118	C/T	
	*qGR-7*	HY15	7	Gm07_2485335	2485335	6.21 × 10^−6^	5.21	4.38	0.22	5.63	−0.145	C/G	
	*qGR-8-1*	JP14	8	Gm08_9896483	9896483	4.44 × 10^−6^	5.35	5.02	0.29	5.32	−0.097	C/T	1
	*qGR-10*	JP14	10	Gm10_4163841	4163841	1.25 × 10^-7^	6.9	6.06	0.11	5.72	0.175	A/G	
	*qGR-13-2*	JP14	13	Gm13_35324537	35324537	1.53 × 10^−6^	5.82	5.02	0.07	5.94	−0.227	G/T	1
	*(QTN13)*	HY15	13	Gm13_35324537	35324537	1.87 × 10^-7^	6.73	5.8	0.07	7.22	−0.265	G/T	1
		Joint	13	Gm13_35324537	35324537	4.23 × 10^−6^	5.37	4.45	0.07	5.44	−0.253	G/T	1
	*qGR-15*	HY15	15	Gm15_11267976	11267976	2.57 × 10^-7^	6.59	5.68	0.07	7.17	0.212	A/T	
	*qGR-18*	JP14	18	Gm18_1336864	1336864	2.76 × 10^-8^	7.56	6.18	0.16	7.68	−0.179	G/T	
	*qGR-20*	HY15	20	Gm20_39515872	39515872	1.69 × 10^−5^	4.77	4.01	0.32	3.96	−0.115	A/G	
NSR	*qNSR-2*	HY15	2	Gm02_46070310	46070310	5.28 × 10^−5^	4.28	4.01	0.14	3.89	−0.144	C/G	
	*qNSR-7*	JP14	7	Gm07_2584000	2584000	1.38 × 10^-7^	6.86	5.8	0.47	7.5	0.117	C/T	
	*qNSR-10*	JP14	10	Gm10_4316320	4316320	1.57 × 10^-7^	6.8	5.97	0.08	7.11	0.242	A/G	1
		HY15	10	Gm10_4316320	4316320	8.89 × 10^-8^	7.05	6.08	0.08	7.61	0.219	A/G	1
	*qNSR-11*	HY15	11	Gm11_36522210	36522210	1.69 × 10^−5^	4.77	4.11	0.08	4.28	−0.161	A/G	
	*qNSR-13-1*	JP14	13	Gm13_35324537	35324537	3.06 × 10^-8^	7.51	6.22	0.07	6.02	−0.328	G/T	1
	*(QTN13)*	HY15	13	Gm13_35324537	35324537	4.26 × 10^-9^	8.37	7.22	0.07	7.04	−0.312	G/T	1
		Joint	13	Gm13_35324537	35324537	1.06 × 10^-9^	8.97	7.33	0.07	6.67	−0.284	G/T	1
	*qNSR-18*	HY15	18	Gm18_59484809	59484809	1.98 × 10^−5^	4.7	3.95	0.08	3.09	0.197	G/T	
	*qNSR-19*	HY15	19	Gm19_48197396	48197396	2.16 × 10^−6^	5.67	5.31	0.1	6.83	−0.147	C/T	
EC	*qEC-3*	JP14	3	Gm03_40169903	40169903	3.86 × 10^−6^	5.41	4.58	0.43	4.85	−229.140	A/G	
	*qEC-7-2*	JP14	7	Gm07_2942021	2942021	9.56 × 10^-7^	6.02	5.19	0.09	4.91	−465.450	A/G	1
		HY15	7	Gm07_2942021	2942021	2.07 × 10^-7^	6.68	5.76	0.09	6.81	−532.980	A/G	1
	*qEC-10*	Joint	10	Gm10_11026230	11026230	2.51 × 10^−5^	4.6	3.81	0.18	4.74	−297.270	G/T	
	*qEC-12*	HY15	12	Gm12_990925	990925	4.90 × 10^−5^	4.31	3.72	0.46	4.55	−192.640	A/G	
	*qEC-13-1*	JP14	13	Gm13_35324537	35324537	3.06 × 10^−5^	4.51	3.69	0.07	4.66	354.477	G/T	1
	*(QTN13)*	HY15	13	Gm13_35324537	35324537	7.99 × 10^-7^	6.1	5.12	0.07	6.37	317.55	G/T	1
		Joint	13	Gm13_35324537	35324537	4.10 × 10^−6^	5.39	5.05	0.07	4.98	307.325	G/T	1
	*qEC-19*	HY15	19	Gm19_35626216	35626216	1.92 × 10^−5^	4.72	4.14	0.08	5.35	400.562	G/T	

^a^ represents the different environments, JP14 represents Jiangpu2014, JP14 represents Huaiyin 2015, Combined represents the Combined-environment based on JP14 and HY15; ^b^ represents the chromosome; ^c^ represents the negative log_10_ transformed *P*-value; MAF represents minor allele frequency; ^d^ 1 represents this QTN can be detected via MLM.

**Table 4:** Functional annotation and positions of nine candidate genes associated with seed-flooding tolerance in the candidate region on Chr.13.

Gene ID	Positon (bp)	Function annotation
*Glyma.13g241900*	35181812-35182687	Dof-type zinc finger DNA-binding family protein
*Glyma.13g243200*	35262813-35264688	NAC domain containing protein
*Glyma.13g244800*	35408963-35410102	Glycine-rich protein
*Glyma.13g246200*	35511992-35512643	F-box family protein
*Glyma.13g246500*	35523224-35524550	transmembrane protein
*Glyma.13g247200*	35571708-35573509	MYB-like DNA-binding protein
*Glyma.13g248000*	35620202-35621661	B-box type zinc finger family protein
*Glyma.13g248400*	35651954-35655747	Leucine-rich Repeat Receptor-like protein kinase
*Glyma.13g249800*	35739290-35742405	Basic helix-loop-helix (bHLH) DNA-binding superfamily protein

## Supplementary Materials

The supplementary materials are available online at https://www.mdpi.com/2073-4425/10/12/957/s1, Table S1: List of 347 soybean lines and the evaluation of GR, NSR and EC in two environments JP14 and HY15, Table S2: Primers used in this study, Table S3: Analysis of variance (ANOVA) of GR and NSR among four lines under different times of flooding treatment, Table S4: SNPs contained in the candidate region on Chr.13, Table S5: Phenotypic data of three selected seed-flooding tolerant and sensitive lines used in qRT-PCR and sequencing analysis, Table S6: Phenotypic correlations (*r*) among GR, NSR and EC in two different environments.
