# Supplementary material for: Identification of QTN and Candidate Gene for Seed-flooding Tolerance in Soybean [Glycine max (L.) Merr.] using Genome-Wide Association Study (GWAS)
Source: Genes (Basel). 2019 Nov 21;10(12):957. doi: 10.3390/genes10120957 (PMC6947551; doi:10.3390/genes10120957)
Supplement: Supplementary file 1 [file genes-10-00957-s001.pdf]

Table S1. List of 347 soybean lines and the evaluation of GR, NSR and EC in two environments JP14 and HY15.

lines	JP14			lines	JP14		
	GR	NSR	EC		GR	NSR	EC
**L001**	0.23	0.13	1024	**L319**	0.96	0.90	793
**L002**	0.26	0.15	1544	**L320**	0.92	0.84	1087
**L004**	0.70	0.55	1215	**L322**	0.27	0.14	1225
**L005**	0.30	0.17	1163	**L323**	0.27	0.10	1579
**L006**	0.43	0.35	1180	**L324**	0.74	0.53	1209
**L007**	0.52	0.46	1379	**L325**	0.70	0.51	772
**L008**	0.50	0.24	1124	**L326**	0.36	0.34	1069
**L009**	0.55	0.36	1393	**L327**	0.21	0.13	1320
**L010**	0.43	0.21	1048	**L328**	0.90	0.63	1089
**L014**	0.29	0.21	1238	**L329**	0.68	0.38	962
**L015**	0.67	0.46	906	**L332**	0.52	0.25	1310
**L016**	0.95	0.88	745	**L334**	0.67	0.23	1199
**L018**	0.96	0.90	154	**L336**	0.66	0.47	931
**L019**	0.65	0.44	1241	**L338**	0.52	0.50	2315
**L022**	0.82	0.79	1088	**L340**	0.72	0.69	958
**L023**	0.69	0.49	1027	**L344**	0.59	0.27	658
**L024**	0.02	0.00	2078	**L347**	0.71	0.39	616
**L026**	0.14	0.13	1704	**L348**	0.88	0.80	597
**L027**	0.67	0.32	1090	**L349**	0.28	0.28	1884
**L028**	0.44	0.20	1643	**L350**	0.50	0.28	799
**L029**	0.36	0.17	1095	**L351**	0.54	0.33	1463
**L031**	0.45	0.26	1638	**L352**	0.86	0.77	910
**L033**	0.26	0.14	1340	**L353**	0.89	0.80	845
**L034**	0.47	0.31	1144	**L355**	0.94	0.83	759
**L035**	0.70	0.47	912	**L356**	0.96	0.90	766
**L036**	0.46	0.23	1112	**L358**	0.13	0.10	1978
**L037**	0.51	0.29	1495	**L360**	0.73	0.43	1082
**L039**	0.48	0.21	1493	**L361**	1.00	0.94	551
**L040**	0.38	0.18	1770	**L362**	0.62	0.22	1163
**L042**	0.90	0.80	886	**L365**	0.26	0.10	1592
**L043**	0.59	0.51	1014	**L366**	0.43	0.32	1196
**L044**	0.19	0.10	1556	**L369**	0.87	0.80	1016
**L046**	0.73	0.50	1219	**L371**	0.06	0.04	1597
**L049**	0.31	0.19	1635	**L373**	0.46	0.20	1113
**L050**	1.00	0.92	582	**L374**	0.20	0.12	1459
**L051**	0.35	0.21	1591	**L375**	0.93	0.83	1140
**L054**	0.73	0.20	1254	**L380**	0.78	0.38	947
**L055**	0.28	0.16	1833	**L383**	0.58	0.23	1105
**L057**	0.25	0.22	1783	**L384**	0.23	0.13	1195
**L058**	0.97	0.84	692	**L386**	0.25	0.11	1052
**L060**	0.94	0.89	629	**L387**	0.58	0.40	1195
**L061**	0.62	0.43	1223	**L388**	0.53	0.27	1063
**L065**	0.04	0.02	1767	**L389**	0.86	0.68	922
**L068**	0.59	0.27	1377	**L390**	0.88	0.84	960

Table S1. List of 347 soybean lines and the evaluation of GR, NSR and EC in two environments JP14 and HY15.

lines	JP14			lines	JP14		
	GR	NSR	EC		GR	NSR	EC
**L071**	0.71	0.57	1099	**L392**	0.92	0.86	1450
**L074**	0.93	0.64	749	**L394**	0.62	0.28	770
**L075**	0.83	0.38	1106	**L395**	0.71	0.65	977
**L076**	0.11	0.10	1553	**L397**	0.42	0.20	1515
**L077**	0.22	0.13	1127	**L400**	0.82	0.64	2236
**L080**	0.28	0.17	1378	**L401**	0.15	0.12	2179
**L085**	0.98	0.50	620	**L402**	0.02	0.00	2175
**L086**	0.90	0.84	712	**L404**	0.22	0.17	1754
**L088**	0.51	0.45	1274	**L405**	0.84	0.24	1444
**L089**	0.85	0.71	912	**L406**	0.67	0.27	1283
**L090**	0.40	0.35	1029	**L408**	0.26	0.17	1796
**L093**	0.24	0.14	1478	**L409**	0.18	0.12	1678
**L094**	0.62	0.54	1274	**L413**	0.88	0.72	676
**L095**	0.76	0.45	923	**L414**	0.13	0.10	2010
**L096**	0.61	0.42	1135	**L415**	0.72	0.59	791
**L098**	0.78	0.55	1202	**L416**	0.78	0.56	1565
**L103**	0.81	0.56	968	**L418**	0.13	0.12	1680
**L104**	0.92	0.71	925	**L419**	0.24	0.15	1289
**L105**	0.42	0.21	1313	**L420**	0.80	0.62	971
**L106**	0.91	0.42	1163	**L422**	0.96	0.93	830
**L107**	0.86	0.43	901	**L423**	0.98	0.71	880
**L109**	0.32	0.13	1046	**L425**	0.63	0.46	521
**L110**	0.67	0.46	1221	**L427**	0.75	0.58	1003
**L111**	0.33	0.17	1318	**L429**	0.40	0.19	1180
**L115**	0.89	0.87	904	**L430**	0.94	0.88	946
**L116**	0.32	0.21	1325	**L431**	0.70	0.64	833
**L117**	0.29	0.18	1333	**L433**	0.57	0.27	809
**L123**	0.59	0.35	1128	**L435**	0.84	0.76	849
**L124**	0.50	0.37	1162	**L437**	0.14	0.13	1571
**L126**	0.17	0.10	1945	**L438**	0.92	0.61	728
**L127**	0.33	0.17	1615	**L441**	0.15	0.11	1842
**L128**	0.55	0.23	1602	**L442**	0.55	0.39	1596
**L129**	0.36	0.22	992	**L443**	0.00	0.00	2840
**L131**	0.85	0.84	1006	**L445**	0.72	0.71	797
**L133**	0.23	0.15	1860	**L447**	0.58	0.40	1171
**L134**	0.33	0.13	1254	**L449**	0.80	0.35	1196
**L135**	0.43	0.31	999	**L452**	0.45	0.19	1744
**L136**	0.33	0.10	1847	**L453**	0.18	0.10	1699
**L137**	0.46	0.19	1510	**L455**	0.29	0.15	1294
**L138**	0.82	0.70	867	**L456**	0.82	0.21	1556
**L140**	0.90	0.87	759	**L457**	0.18	0.00	2012
**L141**	0.21	0.12	1464	**L462**	0.73	0.37	1487
**L143**	0.96	0.94	1154	**L464**	0.91	0.73	983
**L144**	0.34	0.19	1261	**L465**	0.57	0.20	1506

Table S1. List of 347 soybean lines and the evaluation of GR, NSR and EC in two environments JP14 and HY15.

lines	JP14			lines	JP14		
	GR	NSR	EC		GR	NSR	EC
**L149**	0.87	0.74	945	**L466**	0.79	0.51	836
**L152**	0.15	0.12	1625	**L467**	0.70	0.57	1081
**L153**	0.12	0.12	1458	**L468**	0.92	0.83	884
**L154**	0.46	0.19	1582	**L470**	0.38	0.16	1288
**L158**	0.02	0.00	2318	**L473**	0.37	0.00	1022
**L159**	0.47	0.39	1197	**L476**	0.51	0.46	453
**L161**	0.19	0.10	1497	**L477**	0.56	0.10	1175
**L165**	0.90	0.84	814	**L479**	0.51	0.28	1247
**L167**	0.38	0.11	1107	**L482**	0.41	0.38	1281
**L169**	0.65	0.49	1063	**L483**	0.29	0.16	1394
**L171**	0.85	0.79	712	**L487**	0.23	0.19	1791
**L172**	0.69	0.53	1006	**L488**	0.97	0.85	619
**L173**	0.37	0.18	1494	**L489**	0.59	0.14	2250
**L175**	0.73	0.56	1186	**L490**	0.27	0.19	1360
**L177**	0.32	0.29	1284	**L492**	0.92	0.86	792
**L178**	0.26	0.16	1609	**L493**	0.48	0.30	1166
**L180**	0.49	0.18	906	**L496**	0.40	0.19	1249
**L181**	0.70	0.52	1016	**L497**	0.42	0.10	1529
**L183**	0.92	0.81	966	**L498**	0.79	0.71	1302
**L184**	0.40	0.33	1100	**L499**	0.55	0.45	928
**L186**	0.52	0.46	1418	**L500**	0.48	0.19	931
**L189**	0.62	0.45	940	**L501**	0.69	0.62	804
**L191**	0.43	0.35	983	**L503**	0.88	0.65	693
**L195**	0.90	0.84	992	**L505**	0.53	0.48	1144
**L196**	0.03	0.00	2095	**L509**	0.63	0.47	850
**L203**	0.95	0.89	719	**L510**	0.95	0.94	812
**L204**	0.60	0.42	1010	**L511**	0.92	0.90	681
**L206**	0.14	0.10	1566	**L512**	0.75	0.56	923
**L208**	0.32	0.13	2700	**L514**	0.70	0.10	1400
**L209**	0.24	0.10	1861	**L518**	0.41	0.34	935
**L210**	0.66	0.54	1247	**L520**	0.38	0.30	1027
**L212**	0.92	0.84	649	**L521**	0.53	0.50	1614
**L213**	0.47	0.32	1556	**L523**	0.78	0.59	729
**L214**	0.89	0.46	713	**L525**	0.55	0.41	1318
**L216**	0.70	0.42	840	**L526**	0.27	0.16	1284
**L217**	0.11	0.00	2049	**L527**	0.87	0.88	490
**L223**	0.12	0.10	1805	**L528**	0.89	0.79	473
**L224**	0.12	0.10	1854	**L530**	0.22	0.18	1650
**L228**	0.75	0.42	988	**L531**	0.84	0.76	1158
**L230**	0.08	0.00	2580	**L532**	0.36	0.18	1178
**L233**	0.87	0.76	946	**L533**	0.37	0.24	1458
**L234**	0.30	0.12	1353	**L534**	0.61	0.56	1795
**L237**	0.79	0.63	989	**L537**	0.28	0.11	1476
**L239**	0.81	0.80	1140	**L539**	0.43	0.10	1901

Table S1. List of 347 soybean lines and the evaluation of GR, NSR and EC in two environments JP14 and HY15.

lines	JP14			lines	JP14		
	GR	NSR	EC		GR	NSR	EC
**L240**	0.59	0.42	1411	**L540**	0.00	0.00	2475
**L242**	0.84	0.65	805	**L542**	0.26	0.17	2194
**L243**	0.89	0.73	925	**L543**	0.21	0.15	1267
**L245**	0.45	0.30	1329	**L544**	0.19	0.15	2120
**L247**	0.78	0.51	846	**L548**	0.94	0.76	823
**L248**	0.62	0.36	1629	**L549**	0.82	0.53	1210
**L250**	0.55	0.37	1609	**L551**	0.85	0.69	922
**L251**	0.30	0.18	1644	**L552**	0.55	0.15	1204
**L252**	0.95	0.66	777	**L555**	0.76	0.69	982
**L253**	0.87	0.60	744	**L558**	0.70	0.59	1472
**L254**	0.67	0.56	1569	**L559**	0.57	0.35	795
**L255**	0.73	0.48	1874	**L560**	0.62	0.40	1952
**L257**	0.62	0.45	1494	**L561**	0.73	0.63	1060
**L259**	0.62	0.50	1114	**L562**	0.89	0.59	994
**L260**	0.08	0.04	1839	**L563**	0.67	0.39	1166
**L262**	0.76	0.65	866	**L569**	0.72	0.64	1079
**L263**	0.97	0.77	354	**L570**	0.45	0.34	1463
**L266**	0.27	0.20	1472	**L571**	0.83	0.71	951
**L267**	0.67	0.52	1414	**L572**	0.52	0.46	1004
**L268**	0.39	0.19	1328	**L573**	0.17	0.06	1467
**L269**	0.27	0.13	1200	**L574**	0.73	0.41	1112
**L274**	0.24	0.12	2000	**L576**	0.60	0.53	984
**L275**	0.96	0.79	1120	**L577**	0.47	0.10	1482
**L277**	0.26	0.11	1889	**L579**	0.45	0.25	962
**L278**	0.64	0.40	839	**L580**	0.92	0.80	488
**L280**	0.15	0.00	2255	**L582**	0.55	0.19	1582
**L281**	1.00	0.81	514	**L583**	0.85	0.58	926
**L282**	0.42	0.12	1401	**L584**	0.42	0.34	1662
**L285**	0.71	0.61	916	**L586**	0.32	0.21	1479
**L286**	0.68	0.53	1187	**L589**	0.03	0.00	2230
**L288**	0.17	0.12	1387	**L590**	0.43	0.22	1334
**L289**	0.89	0.73	997	**L591**	0.58	0.19	866
**L295**	0.18	0.10	1258	**L593**	0.12	0.11	1576
**L299**	0.65	0.41	1105	**L595**	0.32	0.12	1481
**L300**	0.72	0.51	1437	**L597**	0.32	0.35	1599
**L303**	0.65	0.60	1267	**L598**	0.02	0.00	2390
**L306**	0.75	0.53	1362	**L599**	0.82	0.72	591
**L307**	0.60	0.44	1254	**L600**	0.62	0.27	1247
**L309**	1.00	0.90	375	**L602**	0.03	0.00	1958
**L311**	0.70	0.40	905	**L605**	0.57	0.43	1210
**L316**	0.63	0.40	995	**L606**	0.38	0.24	1849
**L317**	0.38	0.31	1652				

Table S1. List of 347 soybean lines and the evaluation of GR, NSR and EC in two environments JP14 and HY15.

lines	HY15			lines	HY15		
	GR	NSR	EC		GR	NSR	EC
**L001**	0.12	0.04	1253	**L319**	0.76	0.69	953
**L002**	0.16	0.03	1237	**L320**	0.73	0.61	1377
**L004**	0.52	0.25	1491	**L322**	0.06	0.03	1612
**L005**	0.25	0.00	1089	**L323**	0.13	0.08	1733
**L006**	0.25	0.16	1349	**L324**	0.48	0.44	1313
**L007**	0.44	0.34	1125	**L325**	0.46	0.32	1197
**L008**	0.49	0.18	915	**L326**	0.27	0.14	1465
**L009**	0.26	0.14	1537	**L327**	0.12	0.05	1453
**L010**	0.32	0.03	1130	**L328**	0.68	0.56	1271
**L014**	0.18	0.02	1150	**L329**	0.61	0.40	829
**L015**	0.37	0.26	1189	**L332**	0.40	0.19	1508
**L016**	0.84	0.79	831	**L334**	0.41	0.32	1079
**L018**	0.94	0.92	228	**L336**	0.48	0.28	1183
**L019**	0.40	0.25	1522	**L338**	0.21	0.20	1434
**L022**	0.77	0.63	918	**L340**	0.48	0.42	1135
**L023**	0.38	0.22	1307	**L344**	0.53	0.18	771
**L024**	0.02	0.00	2295	**L347**	0.51	0.27	805
**L026**	0.25	0.25	3590	**L348**	0.90	0.79	712
**L027**	0.48	0.28	1566	**L349**	0.09	0.03	2080
**L028**	0.36	0.12	1319	**L350**	0.44	0.26	838
**L029**	0.14	0.00	1367	**L351**	0.35	0.29	1670
**L031**	0.23	0.03	1943	**L352**	0.59	0.45	1030
**L033**	0.32	0.13	1093	**L353**	0.83	0.70	721
**L034**	0.27	0.11	1289	**L355**	0.74	0.59	848
**L035**	0.40	0.34	1225	**L356**	0.78	0.72	857
**L036**	0.33	0.12	1261	**L358**	0.05	0.01	1805
**L037**	0.32	0.09	1573	**L360**	0.52	0.21	1130
**L039**	0.29	0.09	1723	**L361**	0.94	0.90	494
**L040**	0.28	0.01	1632	**L362**	0.54	0.32	912
**L042**	0.81	0.73	844	**L365**	0.07	0.03	1824
**L043**	0.55	0.43	928	**L366**	0.23	0.12	1174
**L044**	0.06	0.02	1467	**L369**	0.77	0.50	1118
**L046**	0.55	0.31	1369	**L371**	0.18	0.00	1740
**L049**	0.19	0.01	1507	**L373**	0.16	0.06	1307
**L050**	0.95	0.93	640	**L374**	0.00	0.00	1609
**L051**	0.24	0.03	1636	**L375**	0.69	0.56	1424
**L054**	0.28	0.28	1761	**L380**	0.46	0.29	1153
**L055**	0.28	0.25	1594	**L383**	0.51	0.22	972
**L057**	0.17	0.04	1648	**L384**	0.18	0.01	1201
**L058**	0.77	0.63	743	**L386**	0.08	0.02	1162
**L060**	0.76	0.67	785	**L387**	0.46	0.27	1071
**L061**	0.54	0.24	1315	**L388**	0.48	0.21	989
**L065**	0.20	0.14	1855	**L389**	0.58	0.46	1031
**L068**	0.46	0.07	1290	**L390**	0.85	0.74	1041

Table S1. List of 347 soybean lines and the evaluation of GR, NSR and EC in two environments JP14 and HY15.

lines	HY15			lines	HY15		
	GR	NSR	EC		GR	NSR	EC
**L071**	0.88	0.86	830	**L392**	0.82	0.69	1217
**L074**	0.83	0.57	952	**L394**	0.46	0.29	961
**L075**	0.59	0.28	1373	**L395**	0.60	0.34	886
**L076**	0.04	0.00	1730	**L397**	0.36	0.12	1324
**L077**	0.03	0.00	1369	**L400**	0.14	0.06	1788
**L080**	0.22	0.08	1250	**L401**	0.02	0.00	2225
**L085**	0.78	0.41	519	**L402**	0.20	0.08	2043
**L086**	0.91	0.83	783	**L404**	0.00	0.00	1995
**L088**	0.48	0.16	1033	**L405**	0.32	0.01	1385
**L089**	0.88	0.60	625	**L406**	0.41	0.23	1394
**L090**	0.31	0.14	872	**L408**	0.08	0.03	1845
**L093**	0.14	0.02	1695	**L409**	0.12	0.00	1535
**L094**	0.59	0.42	1185	**L413**	0.35	0.26	880
**L095**	0.53	0.24	1077	**L414**	0.05	0.00	1547
**L096**	0.58	0.32	973	**L415**	0.73	0.65	595
**L098**	0.78	0.51	1043	**L416**	0.52	0.34	1660
**L103**	0.44	0.21	1365	**L418**	0.07	0.00	1475
**L104**	0.86	0.63	736	**L419**	0.03	0.00	1439
**L105**	0.38	0.13	1043	**L420**	0.64	0.42	1186
**L106**	0.31	0.11	1567	**L422**	0.89	0.68	920
**L107**	0.79	0.31	769	**L423**	0.77	0.69	970
**L109**	0.23	0.03	1149	**L425**	0.59	0.36	642
**L110**	0.43	0.13	1680	**L427**	0.51	0.35	1189
**L111**	0.31	0.29	1398	**L429**	0.14	0.00	1421
**L115**	0.33	0.34	1695	**L430**	0.82	0.66	1007
**L116**	0.25	0.25	3620	**L431**	0.79	0.74	925
**L117**	0.71	0.39	1160	**L433**	0.36	0.04	1018
**L123**	0.32	0.28	1962	**L435**	0.81	0.72	884
**L124**	0.28	0.18	1492	**L437**	0.06	0.04	1493
**L126**	0.06	0.00	1675	**L438**	0.82	0.58	686
**L127**	0.22	0.12	1430	**L441**	0.01	0.00	1760
**L128**	0.21	0.12	1915	**L442**	0.33	0.26	1450
**L129**	0.32	0.14	1105	**L443**	0.17	0.10	2590
**L131**	0.77	0.58	1190	**L445**	0.66	0.49	880
**L133**	0.16	0.09	1650	**L447**	0.52	0.16	947
**L134**	0.33	0.06	1314	**L449**	0.59	0.26	1255
**L135**	0.20	0.06	1324	**L452**	0.44	0.12	1498
**L136**	0.11	0.05	2167	**L453**	0.11	0.05	1530
**L137**	0.46	0.32	1247	**L455**	0.18	0.04	1151
**L138**	0.71	0.58	1263	**L456**	0.26	0.12	1660
**L140**	0.92	0.64	1084	**L457**	0.10	0.02	1830
**L141**	0.02	0.00	1746	**L462**	0.22	0.13	1562
**L143**	0.86	0.73	1043	**L464**	0.68	0.64	913
**L144**	0.28	0.09	1390	**L465**	0.37	0.17	1446

Table S1. List of 347 soybean lines and the evaluation of GR, NSR and EC in two environments JP14 and HY15.

lines	HY15			lines	HY15		
	GR	NSR	EC		GR	NSR	EC
**L149**	0.61	0.49	1181	**L466**	0.71	0.49	788
**L152**	0.03	0.00	1719	**L467**	0.48	0.30	1219
**L153**	0.14	0.04	1507	**L468**	0.81	0.72	814
**L154**	0.24	0.11	1745	**L470**	0.32	0.13	1059
**L158**	0.04	0.02	2470	**L473**	0.28	0.14	989
**L159**	0.24	0.11	1458	**L476**	0.86	0.69	442
**L161**	0.03	0.00	1805	**L477**	0.09	0.01	1190
**L165**	0.87	0.77	703	**L479**	0.26	0.04	1405
**L167**	0.28	0.03	1125	**L482**	0.34	0.13	1160
**L169**	0.43	0.27	1248	**L483**	0.19	0.04	1218
**L171**	0.61	0.50	1085	**L487**	0.21	0.10	1437
**L172**	0.56	0.49	1134	**L488**	0.86	0.78	643
**L173**	0.35	0.09	1225	**L489**	0.38	0.04	1995
**L175**	0.58	0.53	1294	**L490**	0.20	0.00	1216
**L177**	0.27	0.17	1092	**L492**	0.78	0.74	1009
**L178**	0.19	0.08	1519	**L493**	0.33	0.26	1085
**L180**	0.44	0.12	1055	**L496**	0.32	0.19	1189
**L181**	0.83	0.76	1307	**L497**	0.00	0.00	1638
**L183**	0.86	0.63	1045	**L498**	0.69	0.53	1259
**L184**	0.27	0.14	1309	**L499**	0.30	0.13	1030
**L186**	0.38	0.30	1177	**L500**	0.21	0.07	1078
**L189**	0.49	0.26	1153	**L501**	0.59	0.31	813
**L191**	0.35	0.26	997	**L503**	0.61	0.37	795
**L195**	0.72	0.66	1380	**L505**	0.28	0.09	1145
**L196**	0.18	0.10	2315	**L509**	0.58	0.39	765
**L203**	0.91	0.79	869	**L510**	0.73	0.64	931
**L204**	0.52	0.13	1399	**L511**	0.84	0.72	732
**L206**	0.07	0.02	1461	**L512**	0.52	0.37	1039
**L208**	0.23	0.07	2316	**L514**	0.12	0.01	1244
**L209**	0.00	0.00	1262	**L518**	0.31	0.10	891
**L210**	0.47	0.35	1621	**L520**	0.42	0.19	930
**L212**	0.74	0.64	936	**L521**	0.14	0.02	1469
**L213**	0.33	0.24	1477	**L523**	0.49	0.29	921
**L214**	0.79	0.47	1013	**L525**	0.45	0.33	1208
**L216**	0.52	0.24	1061	**L526**	0.20	0.09	1143
**L217**	0.06	0.02	2519	**L527**	0.78	0.67	607
**L223**	0.07	0.02	1680	**L528**	0.82	0.56	617
**L224**	0.00	0.00	2091	**L530**	0.19	0.09	1470
**L228**	0.47	0.19	1307	**L531**	0.87	0.60	1059
**L230**	0.14	0.02	2813	**L532**	0.36	0.19	1069
**L233**	0.61	0.46	1177	**L533**	0.27	0.03	1520
**L234**	0.28	0.02	1433	**L534**	0.21	0.06	1550
**L237**	0.72	0.56	1101	**L537**	0.17	0.02	1313
**L239**	0.59	0.56	1411	**L539**	0.07	0.00	1672

Table S1. List of 347 soybean lines and the evaluation of GR, NSR and EC in two environments JP14 and HY15.

lines	HY15			lines	HY15		
	GR	NSR	EC		GR	NSR	EC
**L240**	0.53	0.37	1517	**L540**	0.22	0.16	1695
**L242**	0.71	0.36	934	**L542**	0.18	0.05	1870
**L243**	0.59	0.51	1342	**L543**	0.20	0.01	1148
**L245**	0.27	0.08	1508	**L544**	0.09	0.03	1892
**L247**	0.70	0.56	735	**L548**	0.81	0.72	940
**L248**	0.41	0.29	1920	**L549**	0.78	0.62	1054
**L250**	0.39	0.28	1775	**L551**	0.62	0.42	1073
**L251**	0.32	0.06	1331	**L552**	0.68	0.08	1064
**L252**	0.74	0.62	1070	**L555**	0.88	0.59	831
**L253**	0.60	0.56	924	**L558**	0.68	0.36	1179
**L254**	0.61	0.48	1153	**L559**	0.67	0.36	942
**L255**	0.42	0.16	2190	**L560**	0.28	0.16	2069
**L257**	0.58	0.39	1610	**L561**	0.54	0.37	1124
**L259**	0.38	0.26	1570	**L562**	0.68	0.60	821
**L260**	0.12	0.04	2370	**L563**	0.37	0.23	1310
**L262**	0.71	0.47	959	**L569**	0.70	0.28	935
**L263**	0.76	0.54	453	**L570**	0.48	0.32	1260
**L266**	0.03	0.00	1915	**L571**	0.82	0.67	814
**L267**	0.58	0.42	1371	**L572**	0.48	0.32	1101
**L268**	0.28	0.07	1421	**L573**	0.25	0.08	1255
**L269**	0.47	0.35	1084	**L574**	0.73	0.35	931
**L274**	0.04	0.02	1601	**L576**	0.32	0.20	1178
**L275**	0.67	0.50	1481	**L577**	0.15	0.08	1101
**L277**	0.04	0.01	2205	**L579**	0.37	0.21	907
**L278**	0.54	0.28	895	**L580**	0.88	0.79	617
**L280**	0.19	0.06	1687	**L582**	0.28	0.11	1820
**L281**	0.66	0.63	760	**L583**	0.62	0.51	1194
**L282**	0.22	0.03	1895	**L584**	0.14	0.03	1950
**L285**	0.69	0.50	1163	**L586**	0.31	0.13	1198
**L286**	0.42	0.29	1539	**L589**	0.23	0.04	1736
**L288**	0.15	0.12	1273	**L590**	0.44	0.28	1165
**L289**	0.59	0.52	1292	**L591**	0.39	0.11	1052
**L295**	0.00	0.00	1597	**L593**	0.02	0.00	1435
**L299**	0.44	0.30	1367	**L595**	0.23	0.10	1380
**L300**	0.49	0.24	1755	**L597**	0.48	0.11	1417
**L303**	0.38	0.04	1595	**L598**	0.02	0.00	2360
**L306**	0.50	0.27	1571	**L599**	0.77	0.61	708
**L307**	0.79	0.66	1820	**L600**	0.39	0.18	1715
**L309**	0.95	0.92	519	**L602**	0.21	0.04	2138
**L311**	0.68	0.32	1072	**L605**	0.63	0.37	965
**L316**	0.41	0.31	1166	**L606**	0.32	0.10	1765
**L317**	0.29	0.13	1920				

Table S2. Primers used in this study.

Genes	Forward (5'-3')	Reverse (5'-3')	Purpose
qRT-*Glyma.13g241900*	GTACCAAAGACTCAGAAGAA	CTTCACCATAGTTATACACACT	qRT-PCR
qRT-*Glyma.13g243200*	TGCTGATAGCTCCTCAAACT	AGAAATCTCCCTAGAAATCT	qRT-PCR
qRT-*Glyma.13g244800*	TTCACCCTCCTTGGCAACCC	ATACCCGGATCCGTATCCTC	qRT-PCR
qRT-*Glyma.13g246200*	ATGTGAAAGTGTCTCCTCGA	GTGGTCGCTTCTCTAATCGT	qRT-PCR
qRT-*Glyma.13g246500*	GGTGCAATTCAGAGGTCTCA	ATCGTTGTTTGAGATTGGCT	qRT-PCR
qRT-*Glyma.13g247200*	AATTCAAGAAGTGCCTGCAT	GCTTAACGCAATCCCATCTT	qRT-PCR
qRT-*Glyma.13g248000*	CTAACAGCGTGGCACGGCAC	TTCAGCCACACCACCATCGT	qRT-PCR
qRT-*Glyma.13g248400*	CTGCCATGTTTGTCAATCAC	ACCACCATCGTCGTTTTCTG	qRT-PCR
qRT-*Glyma.13g249800*	AAAGTACTACTGATTTCGTTG	TCGATGAAGTTGAAGATGAC	qRT-PCR
qRT-*Actin11*	ATGACCCAAATCATGTTTGAGACC	TCAGTTAGATCACGACCAGCGA	qRT-PCR
Glyma.13g244800-cds	ATGCTACAAACTCCACTTCT	TTAGCAATAAGCGATGCACT	gene cloning
Glyma.13g246500-cds	ATGGAGGAGGAGAGCGAGTT	CTAATTGCAACCAATCTCAC	gene cloning
Glyma.13g248000-cds	ATGAGGAAGTGTGAGCTCTG	TTAATCGGAGCCACCTTCTT	gene cloning
Glyma.13g249800-cds	ATGGCTGAGGATTTTCAAGC	CTACCTGCCAATGAGTGCGC	gene cloning
Promoter-*Glyma.13g244800*	GATAGCAGGTGAGACTCGTG	TGTGTTAGTGGAAGGGAAAA	promoter cloning
Promoter-*Glyma.13g246500*	ATTAAACATTCCACCCTCCA	AGAGAATAGGTGATGCTAAGAAA	promoter cloning
Promoter-*Glyma.13g248000*	TTGCAATCTAAAAAACGCTC	TTAAAGAATCTTGAATGATGG	promoter cloning
Promoter-*Glyma.13g249800*	GCCAGTTTATTTAAACTTAAA	GTTGGGGATGTAAGAAAGGT	promoter cloning
GmSFT	AGCCACCATGCTCGAGAT<br>GAGGAAGTGTGAGCTCTG	TGCTCACCATCTCGAGTTAA<br>TCGGAGCCACCTTCTTCAAC	vector construction

Table S3. Analysis of variance (ANOVA) of GR and NSR among four lines under different times of flooding treatment.

Trait	Time	NN88-48	NN86-4	Caidou No.5	Youchu No.4	*F* values	
						Line	Treatment
GR	0d	0.96	0.94	0.96	0.98	1.43^{ns}	/
	2d	0.82	0.85	0.66	0.76	1.87^{ns}	43.14^{***}
	3d	0.64	0.55	0.24	0.18	23.26^{***}	627.49^{***}
	4d	0.15	0.14	0.12	0.05	0.59^{ns}	1530.05^{***}
	5d	0.05	0.06	0.01	0	0.25^{ns}	2644.28^{***}
	6d	0	0.02	0	0	/	
	7d	0	0	0	0	/	
NSR	0d	0.92	0.94	0.88	0.90	1.69^{ns}	/
	2d	0.80	0.73	0.62	0.54	2.15^{ns}	37.25^{**}
	3d	0.56	0.34	0.08	0.12	34.53^{***}	584.17^{***}
	4d	0.10	0.14	0.02	0.02	0.76^{ns}	1729.05^{***}
	5d	0.02	0.04	0	0	0.48^{ns}	3014.36^{***}
	6d	0	0	0	0	/	
	7d	0	0	0	0	/	

^{ns} represents not significant; ** and *** represent significant at p <0.01 and p <0.001.

Table S4. SNPs contained in the candidate region on Chr.13.

SNP name	Position(bp)	SNP name	Position(bp)
Gm13_34829402	34829402	*Gm13_35286347*	35286347
Gm13_34829465	34829465	*Gm13_35324537*	35324537
Gm13_34859160	34859160	*Gm13_35564109*	35564109
Gm13_34860528	34860528	*Gm13_35564167*	35564167
Gm13_34861311	34861311	*Gm13_35568041*	35568041
Gm13_34867304	34867304	*Gm13_35607601*	35607601
Gm13_34867557	34867557	*Gm13_35648515*	35648515
Gm13_34868051	34868051	*Gm13_35648538*	35648538
Gm13_34923404	34923404	*Gm13_35648564*	35648564
Gm13_34958471	34958471	*Gm13_35648582*	35648582
Gm13_34976382	34976382	*Gm13_35655151*	35655151
Gm13_34983680	34983680	*Gm13_35671662*	35671662
Gm13_35006976	35006976	*Gm13_35671834*	35671834
Gm13_35008195	35008195	*Gm13_35690183*	35690183
Gm13_35011421	35011421	*Gm13_35776682*	35776682
Gm13_35123596	35123596	*Gm13_35776687*	35776687
Gm13_35134648	35134648	*Gm13_35776711*	35776711
Gm13_35142844	35142844	*Gm13_35826401*	35826401
Gm13_35194077	35194077		

Table S5. Phenotypic data of three selected each seed-flooding tolerant and sensitive lines used in qRT-PCR and sequencing analysis.

Tolerance	Line	GR	NSR	EC(us/ml)	Env.
Tolerant	L018	0.96	0.90	154	JP14
		0.94	0.92	228	HY15
	L422	0.96	0.93	830	JP14
		0.89	0.68	920	HY15
	L488	0.97	0.85	619	JP14
		0.86	0.78	643	HY15
Sensitive	L217	0.11	0.00	2049	JP14
		0.06	0.02	2519	HY15
	L230	0.08	0.00	2580	JP14
		0.14	0.02	2813	HY15
	L260	0.08	0.04	1839	JP14
		0.12	0.04	2370	HY15

Env., represents environment.

Table S6. Phenotypic correlations (*r*) among GR, NSR and EC in two different environments.

**Trait**	**GR**	**NSR**	**EC**
GR	1	0.92***	-0.66***
NSR	0.90***	1	-0.58***
EC	-0.72**	-0.68***	1

GR, germination rate. NSR, normal seedling rate; The datapoints under and above the diagonal represent *r* values of Jiangpu 2014(JP14) and Huaiyin 2015(HY15), respectively; ** and *** indicates significant level at $P<0.01$ and $P<0.001$.
